# Naturalistic Stimuli in Affective Neuroimaging: A Review

**DOI:** 10.3389/fnhum.2021.675068

**Published:** 2021-06-17

**Authors:** Heini Saarimäki

**Affiliations:** Human Information Processing Laboratory, Faculty of Social Sciences, Tampere University, Tampere, Finland

**Keywords:** emotion, brain imaging, naturalistic stimuli, movies, stories, affective neuroscience, fMRI

## Abstract

Naturalistic stimuli such as movies, music, and spoken and written stories elicit strong emotions and allow brain imaging of emotions in close-to-real-life conditions. Emotions are multi-component phenomena: relevant stimuli lead to automatic changes in multiple functional components including perception, physiology, behavior, and conscious experiences. Brain activity during naturalistic stimuli reflects all these changes, suggesting that parsing emotion-related processing during such complex stimulation is not a straightforward task. Here, I review affective neuroimaging studies that have employed naturalistic stimuli to study emotional processing, focusing especially on experienced emotions. I argue that to investigate emotions with naturalistic stimuli, we need to define and extract *emotion features* from both the stimulus and the observer.

## Introduction

A touching movie, a horror story, or a vivid discussion all evoke strong emotions. What are the neural dynamics of emotional processing in such natural conditions? Affective neuroimaging has traditionally used simple, controlled paradigms, that rely on varying one aspect of a stimulus at the time and often use short, prototypical emotion stimuli such as static images, movie clips, or facial expressions. Naturalistic paradigms have emerged as an approach in neuroscience with great potential for deciphering the neural dynamics in close-to-real-life settings (see, e.g., Sonkusare et al., 2019; Nastase et al., 2020). Compared to controlled paradigms, naturalistic stimuli, such as longer movies and stories, are complex and evoke multimodal processing and strong emotions. However, investigating the neural mechanisms underlying time-varying, higher-order phenomena such as emotions in naturalistic paradigms requires that the mixture of emotion-related signals and their dynamics are extracted and modeled reliably (Simony and Chang, 2020).

Emotions are generally defined as momentary processes caused by internal or external stimuli and leading to automatic changes in multiple functional components, including physiology, behavior, motivation, and conscious experiences (e.g., Barrett et al., 2007; Scherer, 2009; LeDoux, 2012; Anderson and Adolphs, 2014). Compared to semantic and object features that have been successfully extracted from naturalistic stimuli to model dynamic changes in brain activity (Huth et al., 2012, 2016; de Heer et al., 2017), emotion features need to be extracted both from the stimulus and the observer. This results from the abstract nature of emotions: they require higher-order processing of stimuli, integrating information across modalities and processing levels, and emerge at higher areas along the processing hierarchy (Chikazoe et al., 2014). Emotions are not bound only to the sensory features but depend on context, previous experiences, and personal relevance. Thus, for investigating emotions with complex, naturalistic paradigms, a clear framework is necessary for defining what aspect of emotion is studied (see also Adolphs, 2017).

In this review, I describe such a framework based on a consensual, componential view of emotions (for similar approaches, see Mauss and Robinson, 2009; Sander et al., 2018; Vaccaro et al., 2020) and apply this framework to review how naturalistic stimuli have been employed to investigate emotions. Excellent recent reviews introduce methods for naturalistic paradigms in neuroscience (Nastase et al., 2019; Sonkusare et al., 2019) and summarize results across different domains of human cognition (Jääskeläinen et al., 2021). The focus of the current review is on emotions and especially on methodology: how emotions have been extracted from naturalistic, dynamic stimuli, and how emotion-related information has been integrated with brain imaging data. The current paper aims to serve as an introduction for affective scientists interested in embarking naturalistic brain imaging studies. To limit the scope, the focus is on how movies, stories, music, and text have been used to study the neural basis of emotional experiences in combination with functional magnetic resonance imaging. I will focus on studies that have modeled both the emotion-related information and brain activity continuously.

## The Neural Basis of Emotional Experiences

An open question in affective neuroscience is how conscious emotional experiences emerge in the brain (for a review, see LeDoux and Hofmann, 2018). In affective science, emotion is usually broadly defined as a relatively short-lasting state of the nervous system caused by a relevant internal memory or external stimulus and leading to a set of complex changes in behavior, cognition, and body (Mobbs et al., 2019). Emotional experience refers to the subjectively felt, conscious part of emotion (Barrett et al., 2007; Adolphs, 2017). Different emotion theories emphasize either the distinctness of emotion categories (Ekman, 1992; Panksepp, 1992), bodily sensation in emotional experience (Craig, 2002; Damasio and Carvalho, 2013), the functional role of different emotions (Anderson and Adolphs, 2014; Sznycer et al., 2017), or construction of emotions from affective dimensions (Russell, 1980; Barrett, 2017). However, we are far from understanding how neural activity leads to a conscious emotional experience.

**FIGURE 1:**
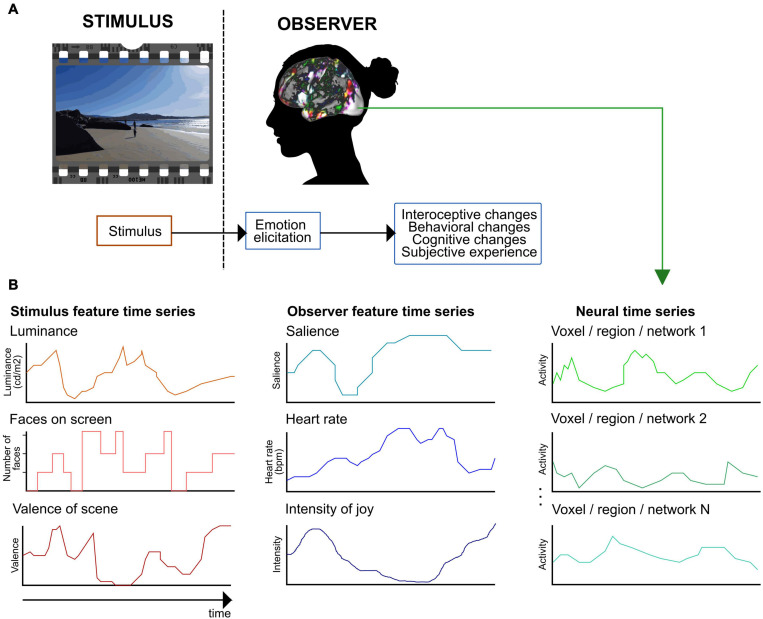
A framework for extracting emotion features in naturalistic paradigms. **(A)** Defining emotion features with a consensual component model of emotional processing (see, e.g., Mauss and Robinson, 2009; Anderson and Adolphs, 2014; Sander et al., 2018). First, the observer evaluates the stimulus’s relevance during an emotion elicitation step. Second, the elicited emotion leads to automatic changes in several functional components. **(B)** Extracting emotion features and example feature time series. With naturalistic paradigms, emotion features can be extracted both from the stimulus and the observer. Stimulus features are related to perceived emotions (here, depicted for a movie stimulus), observer features model experienced emotions. The figure shows examples of potential emotion features and their time series. In the next methodological step, the stimulus and observer feature time series are used to model the neural time series.

Despite their other differences, most current emotion theories agree that emotions are multicomponent phenomena (see e.g., Sander et al., 2018). A consensual, componential view of emotions suggests that they are elicited as a response to personally relevant stimuli and lead to responses in multiple functional components systems (Figure 1A; see e.g., Mauss and Robinson, 2009; Sander et al., 2018; Vaccaro et al., 2020). When relevant emotional input arrives in the brain, several automatic changes take place in split seconds: changes in autonomic nervous system activity including heart rate and respiration, in interoception, in behavior including facial behaviors, voice, bodily behaviors, and actions, in systems controlling behavior, in memories and expectations, and in the interpretation of our current state and that of the environment. These functional components correspond to brain regions or functional networks that all serve different roles in emotional processing and that activate during different emotions (Kober et al., 2008; Sander et al., 2018). For instance, distinct brain networks are responsible for processing somatosensory information, salience, and conceptualization. Brain networks underlying different functional components are activated in an emotion-specific way, leading to emotion-related changes in the state of the central nervous system, visible as different activity patterns of the whole nervous system (Damasio et al., 2000; Kragel and LaBar, 2015; Wager et al., 2015; Saarimäki et al., 2016, 2018). Currently, there is no consensus regarding how emotional experiences evolve in the central nervous system (for a review, see LeDoux and Hofmann, 2018). However, several theories posit that conscious emotional experience emerges as an integration of information from other functional components (Scherer, 2009; Sander et al., 2018). This integration potentially takes place in the default mode network which has emerged as a candidate network for integrating information and holding emotion-specific experiences (Satpute and Lindquist, 2019).

Despite the relevance of functional components in several current emotion theories, most studies have focused on one component at the time. To advance the understanding of how emotional experience emerges and how the different components interact dynamically, studies would need to extract and model variation in multiple components simultaneously. The few studies with such a multicomponent modeling approach show promising results. For instance, studies extracting physiological changes and experienced emotions have revealed candidate brain regions for integrating interoceptive and exteroceptive representations during emotions (Nguyen et al., 2016) or extracted separate neural representations of stimulus’s sensory and affective characteristics (Chikazoe et al., 2014). Multicomponent modeling requires paradigms that elicit multiple aspects of emotional processing. Here, naturalistic stimuli seem promising.

## Emotions in Naturalistic Paradigms

Despite decades of emotion research, we still know relatively little about the neural dynamics of emotions in real-life. Traditionally, emotions have been induced in brain imaging studies using controlled paradigms, such as short videos, music excerpts, or pictures. Controlled studies model emotional responses as static averages across participants and across the whole duration of the stimulus. Emotions are inherently dynamic, and global post-stimulus ratings are not simple averages of the emotion fluctuations during the stimulus (Metallinou and Narayanan, 2013). Similarly, while emotional responses for prototypical, strong stimuli might be consistent across participants, emotions also show sizable individual variation. Thus, while the controlled paradigms are powerful in investigating the neural processes underlying a specific emotional component, such as the recognition of facial expressions, they lack the complicated dynamics of real-life emotional encounters.

Naturalistic stimuli such as longer movies and stories have emerged as a possibility to merge ecologically valid stimuli and laboratory settings to study human cognition (e.g., Hasson et al., 2004; Bartels and Zeki, 2005). Movies and stories are among the most effective stimuli for inducing strong negative and positive emotions in an ethically acceptable way (Gross and Levenson, 1995; Westermann et al., 1996). Thus, naturalistic, complex stimuli are increasingly used in affective neuroscience as they induce reliable, representative neural responses during emotional stimulation (Adolphs et al., 2016). Naturalistic stimuli are vivid, have a narrative structure, and provide a context, thus reflecting the nature of emotional experiences in real life (Goldberg et al., 2014). Importantly, naturalistic stimuli introduce various emotions in a rapidly changing fashion and evoke multisensory processing that preserves the natural timing relations between functional components (De Gelder and Bertelson, 2003). Thus, naturalistic stimuli seem ideal for combining the assets of both ecologically valid emotion elicitation and variation in emotional content, which suits well brain imaging studies with limitations in stimulation time.

The term “naturalistic” is used in neuroimaging to refer to complex stimuli including movies or spoken narratives, even though these stimuli are created by humans rather than occurring naturally (DuPre et al., 2020). Thus, naturalistic paradigms have characteristics that might help us assess how emotions are evoked with the stimuli. For instance, film theory illustrates that movie makers take advantage of local and global narrative cues to induce emotions. Horror films typically use local, visual cues to categorize a character’s behavior and appearance as unnatural or aversive and employ global narrative cues to generate expectations of negative outcome probability for the protagonist (Carroll and Seeley, 2013). On the other hand, empathy toward the protagonist is often induced by close-up scenes displaying a face in distress (Plantinga, 2013). Techniques such as framing, narration, and editing induce strong emotional reactions in viewers, and reciprocally affect the audience’s attention, including how they track actions and events in the film (Carroll and Seeley, 2013). A well-directed movie compared to an unstructured video clip results in more robust synchronization of viewers’ brain activity, suggesting that the stimulus guides their attention similarly (Hasson et al., 2008). Accordingly, the emotionally intense moments of the movies synchronize viewers’ brain activity, supporting the role of emotions in guiding the audience’s attention (Nummenmaa et al., 2012). Parsing the techniques used in creating the stimuli can thus help us better model the emotion features of the naturalistic stimulus.

Each type of naturalistic stimuli also has its unique characteristics, thus providing different tools for studying emotional processing. The current review largely focuses on movies and stories, which are by far the most widely used naturalistic stimuli in affective neuroscience (see Table 1), but other types of naturalistic stimuli such as music and literature have also been used to study the neural basis of emotions (for reviews, see Koelsch, 2010; Jacobs, 2015; Sachs et al., 2015). Movies and stories are forms of narratives where knowledge of emotion elicitation in other domains – including music and literature – is necessary for building complete models of emotional content (see Jääskeläinen et al., 2020). For instance, movies are often accompanied by music as soundtrack or text and stories as dialogue. Music evokes powerful and consistent emotional reactions (Scherer, 2004; Juslin, 2013). The musical soundtrack in movies provides the emotional tone of otherwise neutral or ambiguous scenes (Boltz, 2001; Eldar et al., 2007) and changes experienced emotions in romantic movies (e.g., happy vs. sad soundtrack Pehrs et al., 2013). On the other hand, spoken stories resemble our everyday interaction (Baumeister et al., 2004; Dunbar, 2004) and, in addition to the textual processing, allow investigation of prosody and interaction. Finally, literature can be used to model story comprehension and social representation of the characters (Mason and Just, 2009; Speer et al., 2009; Mar, 2011).

**TABLE 1:** Affective neuroimaging studies with naturalistic stimuli included in the review (organized by stimulus type and year of publication).

Authors study [index]	fMRI sample size	Stimuli (duration hh:mm:ss)	Emotion features	Feature extraction methods	fMRI analysis methods
***Film***					
Moran et al., 2004	25	*Seinfeld* (21:42), *The Simpsons* (22:22)	Humor onset Humor duration	Audience laughter	GLM
Goldin et al., 2005	13	Comedy clips (4:00), *Champ* (2:00), *Stepmom* (2:00), neutral clips (10:00)	Experienced sadness and amusement	Retrospective ratings	Parametric GLM
Nummenmaa et al., 2012	16	Movie segments (∼24:00)	Experienced valence and arousal	Retrospective ratings	Dynamic ISC, seed-voxel correlation analysis
Raz et al., 2012	17	*Stepmom* (8:27), *Sophie’s Choice* (10:00)	Experienced sadness Parasympathetic index (HF-HR)	Retrospective and independent sample (*N* = 39) ratings ECG (*N* = 39)	Intra- and inter-network cohesion index
Sawahata et al., 2013	10	Standup comedy clips (∼45:00)	Humor Viewer’s facial expressions Audience laughter	Simultaneous ratings Simultaneous face camera recording Annotations by independent sample	Decoding models
Naci et al., 2014	12	*Bang! You’re Dead!* (8:00)	Suspense	Ratings by independent sample (*N* = 15)	Parametric GLM
Raz et al., 2014	43, 44	*Stepmom* (8:27), *Sophie’s Choice* (10:00)	Experienced sadness Parasympathetic index (HF-HR)	Retrospective ratings Simultaneous ECG recording	Network cohesion index
Jääskeläinen et al., 2016	18	*The Circus* (9:41), *City Lights* (5:17)	Experienced humor	Retrospective ratings Independent sample (*N* = 18)	Parametric GLM, IS-RSA
Raz et al., 2016	203	*Stepmom* (8:27), *Sophie’s Choice* (10:00), *The X-Files* (5:00), *The Ring 2* (8:15), *Avenge But One of My Two Eyes* (5:21)	Experienced sadness Experienced fear Experienced anger	Retrospective ratings	Network cohesion index
Iidaka, 2017	36	*Mr. Bean* (8 min 9s)	Perceived funniness	Prospective and retrospective ratings	Parametric GLM, dynamic ISC
Schlochtermeier et al., 2017	24	Separation scenes from romantic comedies (∼50:00)	Experienced sadness	Retrospective ratings	Parametric GLM
Young et al., 2017	112	*Irréversible* (10:13)	Heart rate Experienced valence and arousal	Recording Independent sample (*N* = 16)	Intra- and inter-network cohesion index
Jacob et al., 2018	74	Documentary (5:21)	Experienced anger	Retrospective ratings	Dependency network analysis
Nanni et al., 2018	24	*Faces of Death #1* (4:54)	Scene content	Annotations	ICA
Lettieri et al., 2019	15	*Forrest Gump* (∼2:00:00)	Experienced happiness, surprise, sadness, disgust, fear, anger Portrayed emotions from 22 categories	Independent sample (*N* = 12) Independent sample (*N* = 3–12)	Voxel-wise encoding models
Tu et al., 2019	58	Comedy clips (15:00)	Experienced humor	Retrospective ratings	ISC, similarity analysis
Chan et al., 2020	28	Movie trailers (∼40:00)	Experienced valence and arousal Neural pattern for valence and arousal	Independent sample (51) IAPS pattern decoding	Correlation between decoding and ratings time series
Gruskin et al., 2020	112	*Despicable Me* (10:00)	Experienced valence Emotional intensity Visual brightness Auditory loudness Faces on screen	Independent sample (*N* = 20 adults) Absolute value of valence ratings SaliencyToolbox SaliencyToolbox Annotations	Dynamic ISC, fGLS regression
Horikawa et al., 2020	5	Short video clips (∼8:00:00)	Experience of 34 emotion categories Experience of 14 affective dimensions Semantic features Visual features	Independent sample (N = 9-17) Automatic extraction Annotations	Decoding models, voxel-wise encoding models
Hudson et al., 2020	37	*The Conjuring 2* (1:49:17), *Insidious* (1:34:35)	Acute fear onset Experienced fear	Annotations Retrospective ratings and independent sample (*N* = 40)	Dynamic ISC, SBPS
Koide-Majima et al., 2020	8	Short video clips (3:00:00)	Experience of 80 emotion categories	Independent sample (*N* = 166)	Voxelwise encoding models
van der Meer et al., 2020	14	*The Butterfly Circus* (20:00)	Heart rate Pupil dilation Valence of facial expressions Valence of scenes Use of language Mental states	Recordings Recordings Annotations Annotations Annotations Automatic annotation based on Neurosynth	Hidden Markov Models
Brandman et al., 2021	35	*Sherlock* (23:00)	Experienced surprise, intensity and valence Perceived importance Theory of mind Vividness of memory Episodic memory	Retrospective behavioral sampling from an independent sample (*N* = 45)	ISFC
Chang et al., 2021	48	Two episodes of *FNL* (1:30:00)	Experience of 16 emotion categories Facial behavior	Independent sample (*N* = 192) Recordings from an independent sample (*N* = 20)	Hidden Markov Models
***Stories***					
Wallentin et al., 2011	24	*The Ugly Duckling* (20:00)	Experienced valence and intensity Heart rate variability Sound intensity Word frequency Action words	Independent sample (*N* = 53) Simultaneous recording Automatic extraction Annotations Annotations	Parametric GLM
Nummenmaa et al., 2014b	20	Auditory stories (30:00)	Experienced valence and arousal Heart rate, respiration	Retrospective ratings Recording	ISPS, SBPS
Nguyen et al., 2016	20	*Forrest Gump* (∼2:00:00)	Heart rate variability Sound energy envelope	Simultaneous recording Automatic extraction	Parametric GLM, DCM, dynamic ISC
Smirnov et al., 2019	2 speakers, 16 listeners	Autobiographical stories (35:00)	Experienced valence and arousal	Retrospective ratings	Dynamic ISPS
***Music***					
Chapin et al., 2010	14	Frédéric Chopin’s *Etude in E major, Op. 10, No. 3* (∼7:15)	Musical tempo Experienced valence and arousal	Automatic extraction Proactive and retrospective ratings	Parametric GLM
Trost et al., 2015	15	Classical music (Mendelssohn, Prokofiev, Schubert, each 9:00-14:00)	Experienced valence Experienced arousal Acoustic features	Retrospective ratings Independent sample (*N* = 14) Automatic extraction	Dynamic ISC
Sachs et al., 2020	36	*Discovery of the Camp* (8:35)	Experienced sadness and enjoyment Loudness, timbre	Retrospective ratings Automatic extraction	Parametric ISPS, SBPS
***Literature***					
Hsu et al., 2015	24	60 passages from Harry Potter book series	Lexical valence and arousal Experienced valence and arousal	Normative ratings Retrospective ratings	Parametric GLM
Lehne et al., 2015	23	*The Sandman*	Experienced suspense	Simultaneous ratings	Parametric GLM

Taken together, naturalistic paradigms provide means for evoking multicomponent, dynamic emotion processes during brain imaging. However, the less controlled, complex nature of naturalistic paradigms also complicates the modeling of emotion-related information. The critical challenge is that brain activation during naturalistic stimuli reflects multimodal processing, not necessarily related to emotions. The signal reflects various functional component processes happening at different temporal and spatial scales, including but not restricted to sensory processing, evaluation, bodily changes, motor actions, integration, and labeling. Thus, we need to (1) define emotion-related functional components of interest and emotion features reflecting fluctuations in them, (2) extract the emotion features continuously across the stimulus, and (3) integrate the features with brain imaging data to model brain responses underlying different functional components. The current review highlights how these challenges have been solved in affective neuroimaging so far.

## Defining Emotion Features in Naturalistic Paradigms

Modeling the stimulus and task properties is a central challenge with naturalistic paradigms and one that has great potential if solved (Simony and Chang, 2020). Compared to controlled paradigms, naturalistic stimuli allow investigating multiple functional components simultaneously, modeling emotion dynamics at different timescales, and studying individual variation. However, modeling the naturalistic stimulus requires a way to extract time-varying, emotion-related processes. The interpretations based on combining stimulus-driven features with brain imaging data are based on two critical assumptions: first, that the researcher knows which features of the stimulus are driving the brain activity, and second, that these features have been modeled accurately (Chang et al., 2020). Following the terminology in naturalistic studies focusing on visual, object, and semantic features (e.g., Huth et al., 2012, 2016), the term *emotion feature* refers to any emotion-related variable that we can continuously extract from either the stimulus or the observer (Figure 1).

Emotions have several characteristics that guide the definition of features of interest. First, emotions should be modeled across the processing hierarchy. Emotional content leads to modality-specific changes across all functional systems, including separate sensory systems (Miskovic and Anderson, 2018; Kragel et al., 2019). The functional components participating in emotional processing range across the different levels of processing hierarchy, moving from lower, sensory-related processing toward a higher level of abstraction and binding of several lower-level features. Higher-level features in this context refer to socio-emotional representations that are more abstract and consist of integrating different processes including perception, memory, prediction, language, and interoception (Barrett, 2017; Satpute and Lindquist, 2019; Jolly and Chang, 2021). Thus, changes in low-level stimulus features, scene contents, and portrayed emotions are equally crucial for driving emotional responses. However, the low-level features are often treated as nuisance variables, and their effect is removed from the data to guarantee that the brain activity is not solely related to perceptual processing. Yet, the emotional experience depends on integration across all functional components spanning various processing levels including perception (Sander et al., 2018; Satpute and Lindquist, 2019). Features across the processing hierarchy are necessary for a comprehensive model of emotional processing.

Second, emotions result from both the stimulus and the dynamic processes elicited in the observer. Unlike other time-varying features that have been successfully modeled from naturalistic stimuli, including visual, object, and semantic features (Huth et al., 2012, 2016; de Heer et al., 2017), emotion features cannot be modeled only from the stimulus: emotion elicitation also depends on the observer’s evaluation processes. Furthermore, emotional processing includes activating wide-spread functional components within the observer, such as those corresponding to bodily changes and motor planning, which are not directly related to the incoming stimulus but affect the resulting emotional experience (Damasio and Carvalho, 2013; Barrett, 2017; Nummenmaa and Saarimäki, 2019). Thus, accurate modeling of emotion features requires that features are extracted both from the stimulus and the observer.

Third, naturalistic stimuli might lead to various types of emotional processing, which might be necessary to define and model separately (Adolphs, 2017). Emotion features might reflect an emotional state (current state of the central nervous system), emotional experiences (subjectively felt part of the emotional state), semantic processing of emotion concepts (such as fear or love), emotional expressions (automatic or voluntary behaviors associated with the emotional state, including facial behavior), or perception of emotions (understanding the emotional states of other people). For instance, the distinction between *perceived* and *experienced* emotions is critical (Bordwell and Thompson, 2008): *perceived emotions* refer to the emotions portrayed by the characters in the naturalistic stimuli as perceived by the observer, while *experienced emotions* refer to the emotions elicited in the observer. Perceived emotions are guided by low-level features, such as movie soundtrack or the characters’ behaviors (Tarvainen et al., 2014), and elicit automatic socio-emotional processing such as empathy and sympathy, thus leading to emotional experiences. Perceived and experienced emotions often align, but not always: observing a sad facial expression might lead to pity or satisfaction depending on the context and the character (Labs et al., 2015). Furthermore, a horror film or a sad film without a happy end can be enjoyed (Schramm and Wirth, 2010). Thus, the different aspects of emotional processing elicited by the stimulus need to be distinguished and potentially modeled separately.

Once the emotion features of interest have been defined, the next step is to obtain a continuous time series characterizing the dynamic variation of the feature during the stimulation.

## Extracting Emotion Features

Data-driven analyses during naturalistic paradigms often obtain only one, global estimate of stimulus-induced brain activity for the whole duration of the stimulus. This averaging approach has two drawbacks (Bolton et al., 2020): first, it is impossible to know which emotion features drove the estimate, and second, it lowers the sensitivity as temporally localized alignment of feature and brain activity might be concealed by the other time points where feature and brain activity are not aligned. For instance, if we extract the average, stimulus-driven activity across whole emotional movies or narratives, we see mostly primary sensory areas responding. However, adding a continuous, dynamic model of the stimulus features reveals how emotion-related brain networks vary in time throughout the stimulation (Goldin et al., 2005; Nummenmaa et al., 2012, 2014b; Smirnov et al., 2019).

Thus, for accurate modeling of emotion-related variation during naturalistic stimuli, emotion features should be extracted continuously (Figure 1B). The optimal extraction method depends on the feature (Mauss and Robinson, 2009). In affective neuroscience, human ratings and annotations, automatic extraction tools, physiological recordings, and detection of neural activity patterns have been employed to extract continuous emotion features from naturalistic stimuli (see Table 2).

**TABLE 2:** Emotion features in naturalistic paradigms.

Feature	Extraction method	Studies
**Stimulus features**		
**Low-level features**		
Auditory	Automatic extraction (MIDI, MIRtoolbox)	Chapin et al., 2010; Trost et al., 2015; Nguyen et al., 2016; Sachs et al., 2020*
**Object-level features**		
Visual: objects	Automatic extraction (deep neural network for object recognition)	Horikawa et al., 2020
Visual: scene content	Annotations	Nanni et al., 2018
Visual: scene valence	Annotations	van der Meer et al., 2020
Auditory: spoken dialogue	Annotations	van der Meer et al., 2020
Auditory: emotional content	Annotations	Nguyen et al., 2016
Auditory: non-emotional content	Annotations	Nguyen et al., 2016
Semantic: words	Annotations	Schlochtermeier et al., 2017
Semantic: lexical valence	Normative ratings	Hsu et al., 2015
Semantic: lexical arousal	Normative ratings	Hsu et al., 2015
**Portrayed emotions**		
Characters: valence of facial expressions	Annotations	van der Meer et al., 2020
Characters: emotions	Annotations	Lettieri et al., 2019
Characters: empathy	Annotations	Lettieri et al., 2019
Audience: laughter	Annotations	Sawahata et al., 2013
**Observer features**		
**Emotion elicitation**		
Salience	Ratings	Brandman et al., 2021
Expected humor	Annotations	Moran et al., 2004; Sawahata et al., 2013
**Interoception**		
Startle reflex	Annotations (https://wheresthejump.com)	Hudson et al., 2020
Heart rate (HF-HR and LF-HR)	Recording (e.g., ECG, pulse oximeter, pulse plethysmogram)	Wallentin et al., 2011; Raz et al., 2012, 2014; Nummenmaa et al., 2014b; Nguyen et al., 2016; Young et al., 2017; Smirnov et al., 2019*; van der Meer et al., 2020
Respiration	Recording (respiratory belt)	Nummenmaa et al., 2014b; Smirnov et al., 2019*
Pupil diameter	Recording (eye-tracker)	van der Meer et al., 2020
**Neural patterns**		
Mental states	Automatic annotations (Neurosynth)	van der Meer et al., 2020
Valence/arousal patterns	IAPS picture pattern decoding	Chan et al., 2020
**Behavior**		
Facial motion/behavior	Recording (face camera, facial marker motion, and AU analysis)	Sawahata et al., 2013
**Affective dimensions**		
Valence	Ratings	Chapin et al., 2010*; Wallentin et al., 2011; Nummenmaa et al., 2012, 2014b; Trost et al., 2015; Young et al., 2017*; Smirnov et al., 2019; Chan et al., 2020; Gruskin et al., 2020; Brandman et al., 2021
Arousal/emotional intensity	Ratings	Chapin et al., 2010; Wallentin et al., 2011; Nummenmaa et al., 2012, 2014b; Trost et al., 2015; Young et al., 2017*; Smirnov et al., 2019; Chan et al., 2020; Gruskin et al., 2020; Brandman et al., 2021
Other affective dimensions	Ratings	Horikawa et al., 2020
**Emotion categories**		
Amusement	Ratings, audience laughter	Moran et al., 2004; Goldin et al., 2005; Sawahata et al., 2013; Jääskeläinen et al., 2016; Iidaka, 2017; Tu et al., 2019
Anger	Ratings	Raz et al., 2016; Jacob et al., 2018
Enjoyment	Ratings	Sachs et al., 2020
Fear	Ratings	Raz et al., 2016; Hudson et al., 2020
Sadness	Ratings	Raz et al., 2012, 2014, 2016; Schlochtermeier et al., 2017; Sachs et al., 2020
Suspense	Ratings	Naci et al., 2014; Lehne et al., 2015
Basic emotions	Ratings	Lettieri et al., 2019
Other emotion categories	Ratings	Horikawa et al., 2020; Koide-Majima et al., 2020; Chang et al., 2021
**Emotional alignment**		
Synchrony of portrayed and experienced emotions	Correlation of ratings	Smirnov et al., 2019

Asterisk (*) denotes that the feature was used as a control variable in the study.

### Human Ratings and Annotations

Traditionally, emotion features have been extracted using human ratings or annotations. The words ‘ratings’ and ‘annotations’ are used somewhat interchangeably to describe any manually extracted descriptions of the stimuli, but ratings often refer to more subjective features (i.e., observer features) while annotations refer to features that are considered relatively consistent across individuals (i.e., stimulus features). Continuous emotion rating tools originate from the intensity profile tracking approach, which modeled emotion-related changes during stimulation using manual drawings (Frijda et al., 1991; Sonnemans and Frijda, 1994). More recently, various computerized tools have been developed for collecting the ratings (e.g., Nagel et al., 2007; Nummenmaa et al., 2012). Usually, continuous rating tools include moving a slider to indicate dynamic changes in some emotion feature, such as emotional intensity, during the stimulus presentation.

Human annotations have been used to extract various emotion, semantic, and object features from naturalistic stimuli, ranging from low-level visual features to scene contents and portrayed emotions. Manual annotation systems of stimulus-related emotion features have been developed for different social features (Lahnakoski et al., 2012), emotional behaviors, including facial and bodily expressions of emotions (for a review, see Witkower and Tracy, 2019), moments of acute fear onset leading to startle reflex (Hudson et al., 2020), laughter soundtrack during comedy shows (Sawahata et al., 2013), and character’s emotions and empathy (Lettieri et al., 2019).

Human ratings are currently the main method for accessing and modeling observer’s emotional experiences. Most naturalistic studies in affective neuroimaging have collected self-report ratings of some experienced aspect of emotions, including affective dimensions (valence and arousal: Wallentin et al., 2011; Nummenmaa et al., 2012, 2014b; Young et al., 2017; Smirnov et al., 2019; Gruskin et al., 2020; multiple affective dimensions: Horikawa et al., 2020; Koide-Majima et al., 2020) or intensity of categorical emotion experiences (Goldin et al., 2005; Raz et al., 2012; Naci et al., 2014; Jacob et al., 2018; Lettieri et al., 2019; Horikawa et al., 2020; Hudson et al., 2020). Furthermore, also interoceptive observer features including moments of acute fear onset leading to startle reflex (Hudson et al., 2020) have been extracted with ratings. However, extracting experience-related emotion features using ratings has several challenges that affect how reliably the emotion features can be modeled.

Ideally, accurate modeling of experience-related emotion features requires measuring brain activity and ratings from the same participant. However, there are several methodological obstacles for this. First, ratings could be collected either simultaneously with brain imaging or retrospectively. However, collecting ratings during scanning affects neural processing (Taylor et al., 2003; Lieberman et al., 2007; Borja Jimenez et al., 2020). For instance, labeling an emotion requires a conceptual representation of the emotional state, and activating this representation might alter the experience itself (Lindquist et al., 2015; Satpute et al., 2016). On the other hand, the contents of consciousness might only be accessible during the experience itself (Nisbett and Wilson, 1977; Barrett et al., 2007; but see Petitmengin et al., 2013). Collecting ratings retrospectively relies on autobiographical memory and raises concerns of bias due to repetitive viewing effects. However, studies comparing retrospective and simultaneous ratings have shown no differences for the rated intensity of emotion categories such as sadness and amusement (Hutcherson et al., 2005; Raz et al., 2012), although the lack of observed differences might depend on the emotion feature being rated. For instance, while arousal ratings are consistent between repeated presentations of musical pieces, valence shows more variation (Chapin et al., 2010). At the neural level, repetitive viewing of the same stimulus does not alter local activity. However, the network configurations between repetitions differ, suggesting higher-level processing differences between the two viewings (Andric et al., 2016). Moreover, a memory effect on the behavioral similarity between repetitions of ratings cannot be ruled out: the fact that the participants had to perform the rating task already once increases the probability that they later remember what they rated and felt.

Another option is to collect ratings from an independent sample. Here, the assumption is that naturalistic stimuli elicit similar emotional processing across individuals. However, the rating task is often subjective and challenging, which leads to difficulties in obtaining consistent annotations (Devillers et al., 2006; Malandrakis et al., 2011). Uncertainties are caused by motivation, experience with ratings, the definition of emotional attributes, learning curve with the rating tool, shifting the baseline of emotion ratings when becoming more familiar with the dataset, and variation in annotation delays due to individual differences in perceptual processing (Metallinou and Narayanan, 2013). Annotating emotional content is a subjective task and depends on the individual’s perception, experiences, and culture. Continuous ratings increase the complexity of the emotion rating task, requiring a higher amount of attention and cognitive processing (Metallinou and Narayanan, 2013). Furthermore, individual trait differences, for instance, in social desirability or alexithymia, can affect the ratings (Mauss and Robinson, 2009). To ensure the quality, rating data collection requires careful design. Relative ratings are more straightforward to give than absolute ones, so focusing on change rather than absolute values might increase reliability (Metallinou and Narayanan, 2013). Also, instructions should be clear and unambiguous: for instance, when rating portrayed emotions, instructions to empathize with the character compared to detaching from them leads to stronger negative and positive peaks in ratings (Borja Jimenez et al., 2020).

### Automatic Feature Extraction

The rapid developments in automatic feature extraction techniques allow replacing part of the human ratings with automatic tools. Automatic feature extraction usually relies on computer vision or machine learning to find stimulus features that together represent a category. Especially, clearly defined categories might benefit from automatic extraction (McNamara et al., 2017): object categories that were formerly tediously manually annotated can now be extracted automatically from naturalistic stimuli. Going beyond simple low-level sensory features, object and semantic categories, automatic modeling of emotional stimulus content is a central goal in affective computing.

Automatic tools have been developed for extracting both salient low-level visual features (e.g., SaliencyToolbox, Walther and Koch, 2006), auditory features (e.g., MIRtoolbox, Lartillot and Toiviainen, 2007; MIDI Toolbox, Eerola and Toiviainen, 2004), and higher-level features such as faces, semantics, speech prosody, and actions (e.g., pliers, McNamara et al., 2017). In affective neuroimaging of emotions, low-level visual features including brightness (Gruskin et al., 2020) and auditory features such as loudness, tempo, and sound energy envelope (Chapin et al., 2010; Trost et al., 2015; Nguyen et al., 2016; Gruskin et al., 2020; Sachs et al., 2020) have been modeled automatically mainly to control for effects solely driven by sensory features. Object categories such as faces and bodies have been extracted and modeled with automated object recognition tools either from the stimulus (Horikawa et al., 2020) or from the observers (Sawahata et al., 2013; Chang et al., 2021). Sentiment analysis tools have been employed, especially in the study of text-evoked emotions, to model normative valence and arousal of lexical units (Hsu et al., 2015). Finally, tools have recently been developed to automatically extract higher-level prototypical emotion scenes building on ratings of portrayed emotions (Kragel et al., 2019).

Importantly, automatic emotion-related feature extraction often relies on human ratings in the first instance: samples rated by humans are used to train an algorithm to extract features automatically (see, e.g., Kragel et al., 2019). Thus, automatic extraction of emotional content is only as reliable as the original definition of the emotion feature. For instance, automatic recognition of facial expressions (e.g., specific emotion categories from faces) assumes that a correct emotion label was assigned to the facial expressions in the training set, a task that is often underspecified (Barrett et al., 2019).

Automatic feature extraction usually operates at a momentary single-unit level, for instance, on single frames, words, or sounds. However, naturalistic stimuli might elicit emotional processing at various temporal scales. For instance, film makers use methods with different time-scales for enhancing emotional effects (Carroll and Seeley, 2013): the pacing of attention span within short sequences is employed to enhance perceived tension, and global narrative cues build moral expectations of characters or events to drive sustained emotional engagement. Thus, automatic emotion feature extraction currently seems most plausible for prototypical scenes with distinct features that vary in short time intervals.

### Recordings of Peripheral and Central Nervous System Changes

Finally, changes in peripheral and central nervous system activity can be measured directly. Autonomic nervous system activity is often routinely measured in brain imaging studies and can be employed to model bodily changes associated with emotions. On the other hand, brain imaging data also allows automatic recognition of neural patterns associated with the stimulus’s emotional content.

In naturalistic neuroimaging, emotion features related to peripheral nervous system activity have been extracted from recordings of heart rate (Wallentin et al., 2011; Raz et al., 2012; Nummenmaa et al., 2014b; Eisenbarth et al., 2016; Nguyen et al., 2016; Young et al., 2017; Smirnov et al., 2019; van der Meer et al., 2020), respiration (Nummenmaa et al., 2014b; Smirnov et al., 2019), skin conductance (Eisenbarth et al., 2016), and pupil dilation (van der Meer et al., 2020). Since the BOLD signal measured with fMRI also contains physiological noise, recordings of heart rate and respiration are often used to remove noise from brain imaging data (Särkkä et al., 2012). However, autonomous nervous system activity also correlates with emotions and signals bodily changes associated with emotions. Recent studies have demonstrated that combining physiological recordings with other emotion features opens intriguing possibilities for investigating the interplay between bodily and other components of emotions (Nguyen et al., 2016).

Central nervous system activity patterns provide an indirect way to measure various aspects of emotions. For instance, brain states during movie viewing have been automatically labeled as mental states using meta-analytic maps (van der Meer et al., 2020). Given that different emotion categories can be decoded from neural activity (Kragel and LaBar, 2016; Saarimäki et al., 2016, 2018), one possibility for accessing emotional effects during stimulation is to look at the brain of the observer. Continuous pattern decoding tools have been employed, for instance, to demonstrate the utility of brain imaging as an additional predictive measure of human behavior on top of self-report measures (Genevsky et al., 2017; Chan et al., 2019). In affective neuroimaging, a recent application of the technique extracted valence and arousal patterns using an emotional picture viewing task and normative ratings for experienced valence and arousal, and identified corresponding neural activity patterns during a movie viewing task to extract a probability time series for experienced valence and arousal (Chan et al., 2020). In future, pattern decoding approaches seem especially useful for accessing emotion features that are difficult to measure with self-reports.

## Integrating Emotion Features and Brain Imaging Data

Once the emotion features have been extracted, the next methodological step is to combine them with brain imaging data. Multiple methods have been suggested for this purpose. The overall logic in all of them is that the feature time series are used to model the brain response time series to identify the regions where activation varies similarly with the feature. Different approaches focus either on (1) encoding single voxels or regions corresponding to single or multiple features, (2) employing inter-subject synchrony measures to detect stimulus-induced brain activity, (3) decoding feature time series from the activity pattern time series of multiple voxels (multi-voxel pattern analysis), or (4) extracting changes in functional connectivity related to different features.

### Encoding Models

Encoding models in brain imaging use the stimulus to predict brain responses, while the complementary decoding models (see section “Decoding Models”) use the brain activity to predict information regarding the stimulus (e.g., Naselaris et al., 2011). In general, encoding models employ regression to describe how information is represented in the activity of each voxel separately.

By far, the most used approach for combining emotion features to brain imaging data is a univariate approach employing a general linear model (GLM) with parametric regressors (see, e.g., Nummenmaa et al., 2012; Sachs et al., 2020). With a standard univariate regression approach, the feature time series is added as a parametric regressor and applied to all voxel time series to identify the voxels whose activation follows time-varying changes in the feature. Estimates are taken first at an individual level, and group level averages are calculated based on the test statistics. The resulting brain map shows the voxels that follow the feature time course. The traditional GLM approach performs well with models comprising one or few features. Linear regression relies on the assumption that feature vectors are not correlated. However, this is rarely the case with stimulus models comprising multiple features: the feature vectors are linearly dependent of each other, that is, they suffer from multicollinearity. In traditional GLM, multicollinearity is solved by orthogonalizing the feature vectors to avoid unbiased estimates, a practice that is often problematic due to order effects that are difficult to interpret.

Voxel-wise encoding models relying on ridge regression provide a step forward by including multiple features in the regression model simultaneously (e.g., Huth et al., 2012, 2016; Kauttonen et al., 2015). Compared to standard linear regression, ridge regression yields more accurate and unbiased models of data with multicollinearity. Also, voxel-wise encoding models allow direct comparison of multiple feature models (Naselaris et al., 2011). For instance, low-level visual feature models and semantic models could be compared to test whether the voxel represents low-level stimulus properties or more abstract semantic information. A few studies employing voxel-wise encoding models have proven useful, for instance, in comparing the suitability of categorical or dimensional models in explaining brain activity during short, emotional movie clips (Horikawa et al., 2020; Koide-Majima et al., 2020) or in modeling individual emotion rating time series across a full-length feature movie (Lettieri et al., 2019).

### Inter-Subject Synchronization Analyses

Inter-subject correlation analyses include data-driven approaches that assess the similarity of regional time courses from separate subjects exposed to the same time-locked paradigms (Hasson et al., 2004; Jääskeläinen et al., 2008). Inter-subject correlation methods are based on the assumption that the brain signal we measure is a combination of stimulus-driven, idiosyncratic, and noise signal components. If we present the same continuous stimulus to a group of participants and calculate the correlation between subjects’ voxel activation time series, we should be left with the stimulus-driven signal (for recent reviews, see Nummenmaa et al., 2018; Nastase et al., 2019). Thus, inter-subject analyses combined with stimulus and (averaged) observer features can identify the shared emotional responses elicited with naturalistic stimuli.

While standard ISC approaches calculate the inter-subject synchronization across the whole stimulus, dynamic ISC allows tracking the moment-to-moment changes (see, e.g., Bolton et al., 2018). Dynamic ISC requires defining a temporal sliding window of a pair a time series and calculating the average inter-subject correlation for each voxel in each sliding window. Sliding through the whole time series, one can extract the ISC time series and model that with emotion features. However, depending on the window size, the time resolution is compromised. Also, the optimal window size might not be straight-forward to estimate.

Another option is to use inter-subject phase synchronization (ISPS), which provides a continuous measure of inter-subject similarity (Glerean et al., 2012). ISPS corresponds to dynamic ISC, but unlike ISC that relies on correlation as a measure of similarity, ISPS first transforms the signal to its analytic form and then calculates the similarity of phase for each time point without the need for sliding windows. Therefore, it also has the maximum temporal resolution (1TR of fMRI acquisition) and can be used to estimate instantaneous synchronization of regional BOLD signals across individuals. Previous studies showed that ISPS analysis gives spatially similar results to ISC but has better sensitivity (Glerean et al., 2012; Nummenmaa et al., 2014b). Emotion features combined with inter-subject phase synchrony have been used in affective neuroimaging of stories and music (Nummenmaa et al., 2014b; Sachs et al., 2020).

### Decoding Models

Complementary to encoding models, decoding models start with the entire pattern of activity across multiple voxels to predict information regarding the stimulus (Kay and Gallant, 2009). Encoding models and dynamic inter-subject synchronization methods fit the feature time series - be it a single feature or multiple features – to a single voxel’s or region’s activity at a time. Thus, they are univariate, ignoring the simultaneous contributions of several voxels or regions (e.g., Norman et al., 2006). Decoding approaches such as multivariate pattern analysis were developed to overcome this problem. With a dynamic stimulus, multivariate analyses predict the feature model from multiple voxels simultaneously (see, e.g., Sawahata et al., 2013; Horikawa et al., 2020).

For instance, Chan et al. (2020) extracted shared neural patterns for valence and arousal during emotional picture viewing within a region of interest. They applied the resulting classifier for volume-by-volume classification of valence and arousal during movie trailer viewing. Time series for average classification accuracies were extracted and compared to continuous ratings of experienced valence and arousal. Using a different decoding approach, van der Meer et al. (2020) first extracted dynamic brain states during movie viewing using Hidden Markov Models and identified the mental status corresponding to the brain states using Neurosynth’s meta-analysis library.

### Functional Connectivity

Finally, emotion features might also modulate functional connectivity. Once the network time series have been extracted, the methods for combining network time series with emotion features are the same as for voxel or region time series, including encoding, decoding, and inter-subject approaches. In BOLD-fMRI studies, functional connectivity is defined as stimulus-dependent co-activation of different brain regions, usually measured as a correlation between two regions’ time series. Thus, functional connectivity does not necessarily reflect the structural connectivity between two brain regions – two regions might not be structurally connected but still share the same temporal dynamics. In contrast, effective connectivity can be used to investigate the influence one neural system exerts over another (Friston, 2011).

Network modulations of emotion features have been studied with functional connectivity methods including seed-based phase synchronization (SBPS, corresponding to ISPS between regions; Glerean et al., 2012), inter-subject functional connectivity with sliding windows (ISFC, corresponding to ISC between regions; Simony et al., 2016), and inter-network cohesion indices (NCI; Raz et al., 2012). Calculating the connectivity for each pair of voxels is impractical due to the large number of connections this results in. Therefore, functional connectivity is typically calculated as average similarity of voxel timeseries either for selected seed regions of interest or for whole-brain parcellations. To obtain time series of functional connectivity, ISFC and NCI rely on correlations and are calculated with a sliding window approach, while SBPS defines similarity as phase synchrony and can be extracted for each time point directly. Compared to the other two methods, NCI has an additional feature of penalizing higher within-region variance, yielding higher cohesion values for regions that show consistently high correlations (Raz et al., 2012). Affective neuroimaging studies focusing on functional connectivity have revealed emotion-related modulations in various networks (Nummenmaa et al., 2014b; Raz et al., 2014).

Furthermore, the direction of influence between network nodes – effective connectivity - can be investigated with dependency network analysis, which has revealed causal effects between brain regions during emotional states such as anger (Jacob et al., 2018), or with dynamic causal modeling, which has been employed to identify the causal links between regions underlying interoception and exteroception (Nguyen et al., 2016). Dynamic causal modeling can be used to investigate the direction of relations between averaged regions – called nodes – within the functional network, and requires an *a priori* model of the nodes and their directions of influence (Friston et al., 2003). Thus, it cannot be easily implemented in networks including more than a few nodes. On the other hand, dependency network analysis evaluates a node’s impact on the network based on its correlation influence, quantified with partial correlations between the time courses of the node and all other pairs of nodes (Jacob et al., 2016).

Finally, as studies have demonstrated that combinations of functional networks correlate with emotion features, the relationship between network activation and emotion features is likely more complicated than the single-network approaches can reveal (Nummenmaa et al., 2014b; van der Meer et al., 2020).

## Neural Correlates of Perceived Emotion Features: Modeling the Stimulus

**FIGURE 2:**
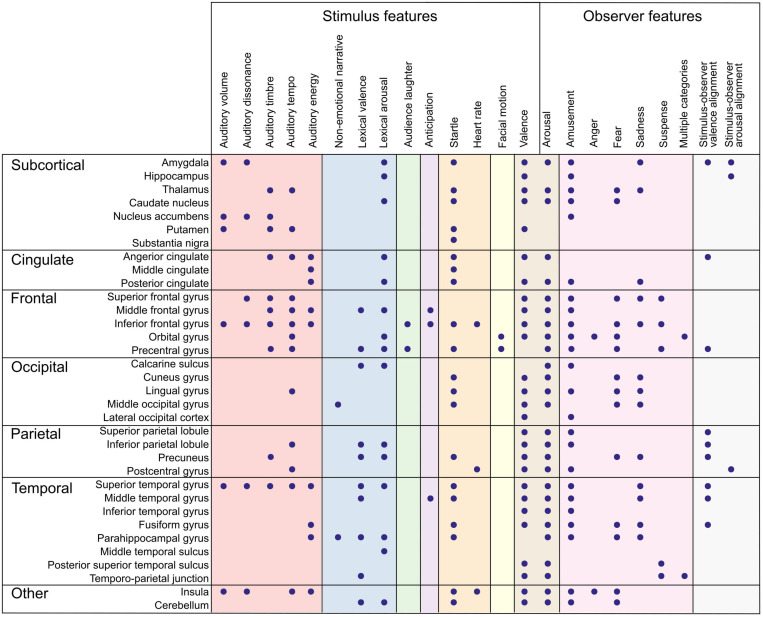
Summary of brain regions correlating with emotion features. Dots denote an observed association between the brain region (rows) and the emotion feature (columns). Color panels denote feature categories (from left to right): low-level auditory features (red), object-level features (blue), portrayed emotions (green), emotion elicitation (violet), interoception (orange), behavior (yellow), affective dimensions (brown), emotion categories (pink), and emotional alignment (gray). Directionality of association and more detailed anatomical locations are listed in Supplementary Table 1.

**FIGURE 3:**
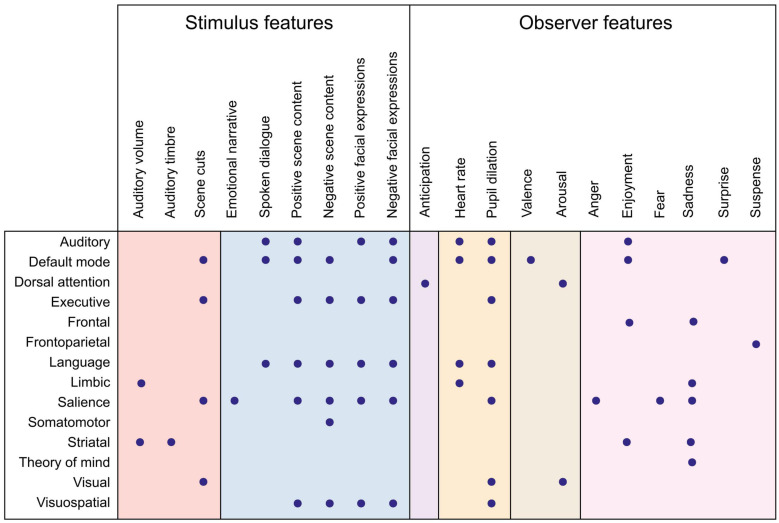
Summary of functional networks correlating with emotion features. Dots denote an observed association between the network (rows) and the emotion feature (columns). Color panels denote feature categories (from left to right): low-level auditory and visual features (red), object-level features (blue), emotion elicitation (violet), interoception (orange), affective dimensions (brown), and emotion categories (pink). Directionality of association and more detailed anatomical locations are listed in Supplementary Table 2.

What has affective neuroimaging with naturalistic paradigms revealed regarding the neural correlates of emotion features so far? As suggested in Figure 1, parsing the emotion features to those related to the perceived emotion (the stimulus) and to the experienced emotion (the observer) helps to clarify what aspect of emotion we are modeling with a specific feature. In the next two sections, I will summarize the results so far, proceeding from lower levels along the processing hierarchy to higher ones, and from stimulus features to observer features. The feature-specific results are presented in Figures 2, 3 and in Supplementary Tables 1, 2.

For perceived emotions, we can distinguish between three types of emotion features in the past studies: low-level stimulus features, object-level features, and portrayed emotions.

### Low-Level Stimulus Features

Low-level stimulus features comprise the visual, auditory, and temporal features that evoke sensory processing in the observer. They can usually be extracted automatically and are often included as nuisance variables to control that the emotion-related activation is not solely related to perceptual features (see Table 2). However, low-level stimulus features might be important for evoking emotions. For instance, affective experiences in music vary with loudness, pitch level, contour, tempo, texture, and sharpness (Coutinho and Cangelosi, 2011).

In affective neuroimaging with naturalistic, low-level auditory features have been extracted and modeled from music and auditory stories (Figures 2, 3). As expected, auditory features are related to activity in auditory regions, but importantly, also in regions traditionally linked with emotions, including subcortical areas (amygdala, thalamus, nucleus accumbens, putamen), anterior cingulate cortex, fronto-parietal regions, and insula (Chapin et al., 2010; Trost et al., 2015; Nguyen et al., 2016; Sachs et al., 2020). Especially, musical tempo increased activation in regions limbic and striatal networks and in executive and somatomotor areas (Chapin et al., 2010). Similarly, behavioral and psychophysiological studies have shown that tempo and loudness in music induce higher experienced and physiological arousal (Schubert, 2004; Etzel et al., 2006; Gomez and Danuser, 2007). Finally, auditory features such as brightness and loudness mediated the correlation between evoked emotions (enjoyment and sadness) and striatal and limbic network activity (Sachs et al., 2020).

So far, low-level visual features have been modeled rarely, but associations of scene switching in movies have been found both with primary visual areas and in default mode and salience networks (van der Meer et al., 2020).

Importantly, some low-level features might directly affect the perceptual pleasantness of the stimulus (Koelsch et al., 2006). For instance, in a study modeling acoustic features from classical music, the activity of nucleus accumbens, an area known to process rewards, was negatively correlated with acoustic features denoting noisiness and loudness (Trost et al., 2015).

Taken together, the regions following dynamic changes in stimulus features include both sensory regions and regions related to higher-level processing. Variation in auditory tempo correlates with somatomotor regions, which might, in turn, correlate with the experienced arousal of the stimulus. The overall stimulus mood, often resulting from variation in low-level stimulus features, also affects the perceived and experienced emotions (Tarvainen et al., 2015). Therefore, removing low-level stimulus features as control variables might remove parts of the emotion-related activation.

### Object-Level Features

Object-level features refer to visual or auditory objects, general scene content, semantic categories, and other intermediate features that bind together multiple low-level sensory features but do not directly reflect the emotions portrayed by the stimulus. For instance, visual object categories such as dogs, houses, and children can be visually present on the screen, identified as separate object categories in the brain, and processed without any reference to emotions. However, object categories often carry emotional connotations and contribute to the contextual emotion cues that affect emotion perception (Skerry and Saxe, 2014). For instance, normative ratings show consistency in perceived affective value of object categories including visual objects (i.e., pictures; Lang and Bradley, 2007; Marchewka et al., 2014; Cowen and Keltner, 2017), auditory objects (i.e., sounds; Bradley and Lang, 2007b; Yang et al., 2018), words (Bradley and Lang, 2007a; Redondo et al., 2007; Stevenson et al., 2007; Yao et al., 2017), and sentences and phrases (Socher et al., 2013; Imbir, 2016; Pinheiro et al., 2017). Oftentimes, annotations of object categories including action words and faces have been included as nuisance regressors (Wallentin et al., 2011; Gruskin et al., 2020). However, also the neural underpinnings of object-level features during emotional stimulation have been investigated.

Object-level features in movies can be distinguished especially within the visual areas in the occipital lobe (Horikawa et al., 2020). Importantly, emotional connotations affect the activation related to visual objects. For instance, aversive visual scene content including injuries evokes activation in the somatomotor network, while different networks (dorsal attention network and default mode network, respectively) are activated prior and after the injury scene (Nanni et al., 2018). Emotional (positive and negative) scenes in movies are associated with high default mode, salience, sensory, and language network activation (van der Meer et al., 2020). Furthermore, general positive scene content has been associated with high executive, sensory, and language network activity and low salience network activity, while negative scene content has been associated with higher default mode and language network activation and lower sensory network activation (van der Meer et al., 2020).

Also, emotional auditory content differs from non-emotional content. Using the audio film Forrest Gump, Nguyen et al. (2016) extracted both the original, emotionally salient film soundtrack containing background music and dialogue and an audio commentary track with neutral prosody describing the visual content of the movie. The emotionally salient film soundtrack strongly activated the salience network. However, the commentary correlated with middle occipital gyrus and parahippocampal gyrus activation, probably reflecting the narrated environmental scene descriptions and evoking spatial imagery.

Semantic features in movies are represented especially within brain networks related to auditory, language, and executive processing in lateral frontal, temporal, and parietal regions (Horikawa et al., 2020). Also in the case of semantic features, affective characteristics at various levels affect the feature representation, and can be extracted with sentiment analysis (for a review, see Jacobs, 2015). Word-level affective value based on normative ratings of valence and arousal predicts the BOLD response to affective text passages in emotion-associated regions more strongly and widely than the experienced emotion ratings from the same passages. Besides the traditional emotion-related regions, such as amygdala and cingulate cortices, effects of normative lexical valence and arousal were also found in this study in regions associated with situation model building, multi-modal semantic integration, motor preparation, and theory of mind, suggesting that it is not solely the early emotion areas that activate for words with different affective values (Hsu et al., 2015).

### Portrayed Emotions

Portrayed emotions have been extracted mainly with manual annotations (Ekman and Oster, 1979; Lahnakoski et al., 2012; Witkower and Tracy, 2019). Emotion cues used in labeling portrayed emotions include, for instance, facial behaviors (e.g., frowning, Volynets et al., 2020), bodily behaviors (e.g., Witkower and Tracy, 2019), verbal and vocal cues (e.g., trembling voice, Ethofer et al., 2009), actions, and contextual cues (Skerry and Saxe, 2014). Perception of emotions operates at an abstract level regardless of the stimulus modality: both inferred (from contextual descriptions) and perceived (from facial expressions) have shared neural codes (Skerry and Saxe, 2014).

Social feedback alters emotional responses (Golland et al., 2017). Thus, another aspect to consider is the third-person view of the portrayed emotions: the depicted reaction of the audience or another character. For instance, in comedy clips, audience laughter might guide observers’ emotional reactions (Moran et al., 2004; Sawahata et al., 2013). Similarly, movies might portray other people’s reactions to the character’s emotion, potentially affecting also the emotion elicited in the observer. The third-person effect has been approached by categorizing portrayed emotions either to self-directed emotions (i.e., how the character feels) or other-directed emotions (i.e., how the character feels for other characters) (Labs et al., 2015; Lettieri et al., 2019). For instance, Lettieri et al. (2019) showed that ratings of subjective experience share 11% of the variance with the self-directed emotion attribution model and 35% with the other-directed model. Thus, it seems that empathic responses between the observer and characters align better than portrayed and experienced emotions.

Furthermore, emotional alignment and misalignment are important aspects to distinguish. Studies using naturalistic stimuli for emotional induction sometimes assume that we automatically align with the characters’ emotions. Accordingly, similar brain activity might underlie affective judgments of experienced emotions and emotions portrayed by the protagonist (Schnell et al., 2011). Also, higher emotional alignment is related to activation within the mentalizing networks and regions associated with the mirror neuron system (Zaki et al., 2009). However, misalignment between portrayed and experienced emotions is equally possible and might be relevant in naturalistic stimuli where longer-term contextual cues modulate the interpretation of characters’ motivation. For instance, a happy expression on the villain’s face might lead to misalignment. Also, the stimulus’s general mood is not necessarily congruent with the emotions expressed by the characters (Plantinga, 1999).

## Neural Correlates of Experienced Emotion Features: Modeling the Observer

The other aspect of emotions we can parse from naturalistic stimuli relates to the emotional processing evoked in the observer. As the consensual, componential approach to emotions suggests, emotions evoke both unconscious and conscious changes ranging from emotion elicitation to interoceptive and motor changing and to integration of overall activation into conscious emotional experiences and labeling the emotional experience using emotion concepts (Scherer, 2009; Satpute and Lindquist, 2019). The neural activation during naturalistic emotional stimuli reflects all these functional components.

Thus, for emotional processing in the observer, we can identify five types of emotion features in the past studies, each corresponding roughly to a different functional component: emotion elicitation, interoception, behavior, affective dimensions, and emotion categories.

### Emotion Elicitation

When sensory input arrives in the brain, it is evaluated for personal relevance (see, e.g., Ellsworth and Scherer, 2003; Okon-Singer et al., 2013). This process - emotion elicitation – includes several overlapping processes, including saliency detection and cognitive appraisals (for a review, see Moors, 2009). Emotion elicitation pairs the incoming stimulus with individual’s prior experiences, beliefs, and values (Scherer, 2009). The salience network, consisting of anterior cingulate cortex and anterior insula, is probably the most well-established functional brain network associated with emotion elicitation (Menon, 2015; Sander et al., 2018). However, salience detection associated with the salience network is only one aspect of emotion elicitation, and also other appraisal features have proven useful in distinguishing between brain activity underlying different emotion elicitation processes (Skerry and Saxe, 2015; Horikawa et al., 2020).

Stimulus salience effects can be extracted either automatically or using ratings, which potentially depend on partly different processes related to salient sensory features and more cognitive detection such as ratings for relevance, respectively. While biologically salient stimuli usually activate the salience network (Sander et al., 2003; Pessoa and Adolphs, 2010), relevant goal-directed hand actions during a movie have been linked to activity in the posterior parietal cortex (Salmi et al., 2014).

Anticipation, or expectancy, is another appraisal feature that has been successfully modeled in affective neuroimaging. For instance, activity in the dorsolateral prefrontal cortex, inferior frontal gyrus, ventromedial cortex, and temporal areas predict humor onset already a few seconds before the amusing episode started (Moran et al., 2004; Sawahata et al., 2013). In a study with aversive stimuli, the dorsal attention network was active when scenes with injuries were anticipated (Nanni et al., 2018).

Importantly, individual appraisals reflect different perspectives on the same stimulus (Okon-Singer et al., 2013). Thus, modeling appraisals might be essential for understanding individual differences in emotion elicitation. For instance, perspective-taking studies have shown that the observer’s perspective modulates the neural responses already in the visual cortex (Lahnakoski et al., 2014). Similarly, visual cortex activation differs between experienced emotions and might partially relate to the role of appraisals in guiding the sensory activation underlying different emotions (Kragel et al., 2019). While affective neuroimaging studies rely largely on average responses, the individual effects of appraisal processes remain poorly understood. Naturalistic paradigms compared with feature modeling provide a way forward in this domain.

### Interoception

Interoception refers to the central processing of physiological signals and allows perceiving the bodily state both consciously and unconsciously (Khalsa et al., 2018). Interoceptive information regarding bodily changes plays a vital role in emotional experiences according to several emotion theories (e.g., Critchley et al., 2004; Damasio and Carvalho, 2013; Seth, 2016; Barrett, 2017). Naturalistic stimuli, including movies, stories, or merely imagining an emotional event, induce strong physiological reactions (Carroll and Seeley, 2013; Nummenmaa et al., 2014a; Saarimäki et al., 2016). While the physiological changes related to emotions are usually measured externally, I will adopt the term interoception to emphasize that part of the brain activity patterns related to emotional experiences reflect the central processing of physiological signals.

Autonomic nervous system activity, including heart rate and respiration rate, is routinely measured during functional magnetic resonance imaging to account for physiological “noise” in brain activity (Chang et al., 2008). However, the physiological measures also correlate with emotion features, and reflect the variation in bodily changes, which in turn constitute an important functional component contributing to the overall experienced emotion. Especially, the experienced arousal or emotional intensity has been linked with increases in heart rate variability (Wallentin et al., 2011; Young et al., 2017). Heart rate is associated with variation in salience and default mode network activity (Young et al., 2017). In a seminal naturalistic study, the insula was identified as a hub for integrating sensory and interoceptive information during emotional moments of an audio narrative consisting of dialogue and music (Nguyen et al., 2016).

The neural basis of different autonomic responses is not unitary. For instance, during stressful situations, heart rate and skin conductance level are associated with partly common and partly unique brain activity patterns (Eisenbarth et al., 2016). For emotions, this means that while one cannot assume that different emotion categories have consistent changes in one measure (Siegel et al., 2018), the overall sum over all physiological measures might differ between emotions. Both heart rate and skin conductance responses correlated with activity in the anterior cingulate, ventromedial prefrontal cortex, cerebellum, and temporal pole, while several frontal regions were more predictive for either heart rate or skin conductance (Eisenbarth et al., 2016). In another study, larger pupil size strongly correlated with scene luminance, but also with brain states of high executive, sensory, and language network activation, and high default mode network and salience network and low executive network activation (van der Meer et al., 2020).

### Emotional Behaviors

Brain imaging studies of emotions often show activity in motor regions (Saarimäki et al., 2016, 2018). These patterns probably reflect the emotion-related behaviors that are automatically activated and inhibited in the observer. On the other hand, behavioral changes are an essential part of the emotional state and emotional experience (Sander et al., 2018). The observer’s emotional behaviors include the same behaviors as earlier listed for emotions portrayed by the characters: vocal characteristics, eye gaze, facial behaviors, and whole-body behaviors (Mauss and Robinson, 2009). Following Mauss and Robinson (2009), I will use the term ‘behavior’ instead of ‘expression’ as the term ‘expression’ implies that emotions trigger specific behaviors, while the literature is inconsistent in this (Barrett et al., 2019).

While brain imaging setups restrict most explicit emotional behaviors, the few studies measuring emotional behaviors have focused on changes in facial behaviors (Sawahata et al., 2013; Chang et al., 2021). For instance, Sawahata et al. (2013) showed that facial motion related to amusement during comedy film viewing could be decoded from motor, occipital, temporal, and parietal regions. Furthermore, Chang et al. (2021) showed that changes in facial behaviors were reflected in the activity of ventromedial prefrontal cortex.

Naturalistic stimuli are not interactive, implying less need for communicative behaviors. However, it is possible that the brain activity patterns measured with affective stimuli reflect also automatic emotional behaviors and inhibition, as suggested by the automatic facial behaviors evoked during emotional movie viewing (Chang et al., 2021). For instance, displaying and observing facial behaviors activate partly overlapping brain regions (Volynets et al., 2020). Similarly, observing actions in movies and real-life automatically activates overlapping motor regions (Nummenmaa et al., 2014c; Smirnov et al., 2017).

### Affective Dimensions: Valence and Arousal

Dynamic ratings of experienced valence and arousal serve as emotion features in several studies (Nummenmaa et al., 2012, 2014b; Smirnov et al., 2019). Valence and arousal refer to the dimensions of pleasantness–unpleasantness and calm–excited, respectively, observed initially in studies comparing emotional experiences (Russell, 1980). However, valence might not be a unitary concept as previously thought, and it might be found and evaluated at different processing levels (Man et al., 2017). For instance, representations of valence and arousal in pictures and movies have been found in lower (LOC; Chan et al., 2020) and higher areas along the processing hierarchy (OFC; Chikazoe et al., 2014). Especially, valence-related evaluations can focus on appraisal-level stimulus features, to interoceptive sensations, or to the emerging emotional experience resulting from the integration of multiple functional components. Similarly, the unitary concept of arousal has been questioned (Sander, 2013): arousal can refer to, for instance, bodily changes such as muscle tension or to cortical states of vigilance (e.g., Borchardt et al., 2018). Thus, arousal is closely linked with interoceptive variation during emotional stimuli. Therefore, valence and arousal are best understood as broad dimensions and variation in them might be linked to emotional processing in different functional components.

Valence has been associated with activity in a wide range of regions, including subcortical regions, cingulate cortex, insula, and most cortical areas ranging from early sensory areas to areas associated with higher-level processing (Figures 2, 3 and Supplementary Tables 1, 2; Wallentin et al., 2011; Nummenmaa et al., 2012, 2014b; Trost et al., 2015; Smirnov et al., 2019; Chan et al., 2020; Gruskin et al., 2020). Such widespread activity further suggests that the experienced valence ratings reflect several changes in underlying components, leading to an umbrella label of valence. Supporting this view, subcortical responses including decreases in amygdala and caudate activity during music were driven mainly by low-level, energy-related musical features, including root mean square and dissonance (Trost et al., 2015). Besides low-level stimulus features, valence also correlates with other emotion features. Especially, experienced negative valence during emotional stories correlates with increased heart rate and respiration rate; however, this effect can be difficult to distinguish from arousal, as valence and arousal time series in this study were highly correlated (Nummenmaa et al., 2014b).

Experienced arousal has been linked with wide-spread brain activity spanning most of the brain and, especially, sensory and attention-related brain regions (Figures 2, 3 and Supplementary Tables 1, 2; Wallentin et al., 2011; Nummenmaa et al., 2012, 2014b; Trost et al., 2015; Smirnov et al., 2019; Chan et al., 2020; Gruskin et al., 2020). Similarly to valence, low-level stimulus features can drive arousal. For instance, the correlation between experienced high arousal and activity in subcortical (amygdala, caudate nucleus) and limbic (insula) regions was driven by low-level, energy-related features in music (Trost et al., 2015). In line with this, physiological arousal during movies correlates both with experienced arousal and with activity in the salience network, linking arousal to detection of emotionally relevant stimulus segments (Young et al., 2017). Arousal also varies with autonomous nervous system activity, supporting the role of arousal as a physiological component (Mauss and Robinson, 2009). Subjectively rated arousal peaks coincide with increased heart rate during movie viewing (Golland et al., 2014; Young et al., 2017). However, experienced arousal only correlated with respiration rate but not with heart rate when using emotional stories with neutral prosody (Nummenmaa et al., 2014b). Furthermore, continuous ratings of experienced arousal are associated with increased activity in somatosensory and motor cortices and activity in frontal, parietal, and temporal regions and precuneus (Smirnov et al., 2019).

Affective dimensions also dynamically modulate functional networks. In a study using positive, negative, and neutral stories, higher experienced emotional arousal and negative valence were correlated with widespread increases in the connectivity of frontoparietal, limbic (insula, cingulum), and fronto-opercular (motor cortices, lateral prefrontal cortex) regions for valence and those of subcortical, cerebellar and frontocortical regions for arousal, while connectivity changes due to positive valence and low arousal were minor and involved mostly subcortical regions (Nummenmaa et al., 2014b).

Taken together, affective dimensions including valence and arousal are linked to wide-spread activity changes across the brain. Thus, parsing their effects in specific functional components by extracting and modeling other, more low-level emotion features is necessary for understanding what aspect of emotional processing the dynamic ratings of valence and arousal are measuring.

### Emotion Categories

Besides affective dimensions, another option for directly extracting the emotional experiences is to collect ratings of experienced intensity for various emotion categories, such as joy or fear. Based on previous results in multivariate pattern recognition studies, experienced emotion categories modulate brain activity across a wide range of regions (Saarimäki et al., 2016, 2018). Furthermore, if the experience of emotions emerges from the integration of activity across functional components, activation in component-specific regions might also follow the variation in experience. Thus, compared to other emotion features, experienced emotions should show modulations in multiple brain regions. For instance, time-series of experienced joy might correlate with lower-level feature time series such as brightness. Thus, the activation patterns we see during episodes of joy might also reflect lower-level sensory processing.

Most studies with categorical emotions have focused on modeling the dynamic variation of intensity for one emotion category at a time. Thus, the stimulus materials in affective neuroimaging studies have often targeted one emotion category (e.g., horror movies to elicit fear in Hudson et al., 2020; comedy clips to elicit amusement in Iidaka, 2017; or separation scenes to elicit sadness in Schlochtermeier et al., 2017). Emotion categories that have been studied with naturalistic stimuli include amusement (Moran et al., 2004; Sawahata et al., 2013; Iidaka, 2017; Tu et al., 2019), sadness (Goldin et al., 2005; Schlochtermeier et al., 2017; Sachs et al., 2020), enjoyment (Sachs et al., 2020), fear (Hudson et al., 2020), suspense (Naci et al., 2014; Lehne et al., 2015), and anger (Jacob et al., 2018). Overall, as expected, categorical emotions lead to wide-spread brain activity and connectivity changes similarly to valence and arousal (see Figures 2, 3 and Supplementary Tables 1, 2). The emotion-specific differences might result from differential activation in underlying functional components which results in differences in the consciously experienced emotion.

The experienced intensity of multiple emotion categories has recently been modeled simultaneously, allowing the investigation of shared and unique responses across categories. For instance, Lettieri et al. (2019) extracted ratings of experienced happiness, sadness, disgust, surprise, anger, and fear during a full-length movie. Associations with rating time series were found in frontal regions, motor areas, temporal and occipital areas, and especially in temporo-parietal junction, which showed peak activation across all emotion categories. Temporo-parietal junction has been identified as a hub for various types of social processing in movies (Lahnakoski et al., 2012). Accordingly, the categorical emotion ratings were in Lettieri et al. (2019) were mainly correlated with social, other-directed emotions portrayed by the characters.

Interestingly, neural correlates of experienced emotion categories might vary depending on the content of the movie. Raz et al. (2014) found that unfolding sadness-related events during a movie led to correlations between limbic network and sadness ratings, while sadness-related discussions of future events in a movie led to correlations between theory-of-mind network and sadness ratings. This demonstrates the value in carefully evaluating and modeling the content of stimuli.

Dimensional emotion theories posit that emotion categories can be represented along a few dimensions. Thus, if dimensions such as valence and arousal are enough for explaining the variance in emotional experiences, collecting data from multiple emotion categories might not bring additional value. To test this, Horikawa et al. (2020) compared a dimensional model (14 features representing affective dimensions) and a categorical model (34 features representing the intensity of emotion categories). They found that the categorical model explained the variance in brain activity better than the dimensional model.

Taken together, the wide-spread brain activity changes associated with emotion categories support their role in integrating activation across functional components. Especially, studies with more fine-grained analysis between experienced and portrayed emotions (Lettieri et al., 2019) and parsing the same emotion category to different appraisals (Raz et al., 2014) suggest that more detailed modeling of stimulus and observer features is necessary for understanding how the category-specific brain activity emerges.

## Future Directions

### Temporal Dynamics of Emotion Features

Emotions are momentary by definition (see, e.g., Frijda, 2009; Mulligan and Scherer, 2012). Several emotion theories involve changes that evolve across time and components dynamically affecting each other (e.g., Scherer, 2009; Gross, 2015). Despite the growing theoretical interest in the dynamics of emotions, the empirical studies regarding the dynamics of neural circuitries in human neuroscience remain sparse (for a review, see Waugh et al., 2015). Yet, the few studies that have extracted and directly compared time series from multiple brain regions suggest that different functional components might have different time-scales during emotional stimulation (Sawahata et al., 2013).

The time-varying feature time series have the potential of informing us regarding the neural dynamics of emotions. The two most prominent sources of emotion dynamics are their degrees of explosiveness (i.e., profiles having a steep vs. a gentle start) and accumulation (i.e., profiles increasing over time vs. going back to baseline; Verduyn et al., 2009). Similarly, movies elicit two types of affective responses: involuntary, automatic reflexive responses that produce intense autonomic responses and grab our attention, and more cognitively nuanced emotional reactions (Carroll and Seeley, 2013). In a recent study, Hudson et al. (2020) modeled acute and sustained fear separately. They found differences between brain regions underlying acute (explosive) and sustained (accumulated) fear. Sustained fear led to increased activity in sensory areas while activity in interoceptive regions decreased. Acute fear led to increased activity in subcortical and limbic areas, including the brainstem, thalamus, amygdala, and cingulate cortices. Dynamic approaches have also proven useful in detecting the brain regions and networks responsible for processing at consecutive stages of evolving disgust (Nanni et al., 2018; Pujol et al., 2018), sadness (Schlochtermeier et al., 2017), and humor (Sawahata et al., 2013). For instance, using the temporal dynamics of humor ratings, predicting the upcoming humor events on a volume-by-volume basis was possible before they happened (Sawahata et al., 2013), and cumulative viewing of sad movies increased activation in midline regions including anterior and posterior cingulate and medial prefrontal cortex (Schlochtermeier et al., 2017). Thus, parsing the functional components using emotion feature models allows investigation of temporal variation between components, and opens new possibilities for naturalistic neuroimaging of emotions.

Also, different emotional states might vary in their temporal dynamics. For instance, while changes in cortical arousal compared to baseline were visible for resting-state following emotional narratives evoking various emotions, their temporal dynamics differed from each other (Borchardt et al., 2018). Thus, different emotions have different temporal scales: some might take longer to recover from, and some might take longer to develop. However, currently, most studies with emotion induction assume that all emotions develop at the same pace. Naturalistic stimuli and data-driven methods might provide insight for this: by investigating the moments in time when synchronization (for instance, with inter-subject correlations) becomes similar or different across different emotions, one could gain further insight into what aspect of the stimulus is processed in a given region (Borja Jimenez et al., 2020).

### Individual Differences in Emotional Processing

A central problem in affective neuroscience relates to ignoring individual differences. Often, studies have assumed that similar emotions are elicited in all participants, implying that the idiosyncratic part of the BOLD signal is uninformative of the phenomenon of interest. However, this averaging approach ignores the considerable individual variation in emotional experiences and in the functioning of different components, including emotion elicitation, bodily changes, and behavior. For instance, brain activity patterns underlying different emotions are more consistent within individuals than between individuals, demonstrating individual variation either spatially or in emotional processing in general (Saarimäki et al., 2016). Furthermore, variation in arousal modulates inter-individual variability in functional connectivity (Jang et al., 2017). With prototypical stimuli, emotion elicitation likely varies less between participants, as the strong content guides participants’ attention similarly (Carroll and Seeley, 2013). However, especially more subtle emotional stimuli might be interesting from an individual differences perspective.

Similarly, studies investigating individual differences in rating behavior have demonstrated subject-specific delays between an emotional event and its annotations (Metallinou and Narayanan, 2013), suggesting individual variation in neural processing patterns and dynamics. One way to study individual variation in processing time is to extract variation in gaze patterns. Pfeiffer et al. (2020) extracted individual timings from fixation to words measured with eye-tracking and combined these with normative word valence ratings during a naturally paced reading task. This approach allowed the investigation of how emotionally laden words affect individual processing dynamics.

One way to accommodate individual differences is to model the time series individually. Individual models would need to cover quantitative and qualitative differences between participants and account for noise in individual ratings. For instance, Chan et al. (2020) used a combination of individual, static ratings, and continuous online ratings from an independent sample. One further possibility for overcoming problems with average models is identifying subgroups of participants that experience similar emotions and modeling time series for the groups separately.

Another approach to individual differences is investigating the time points in naturalistic stimuli when emotional processing either converges or diverges. For instance, pairwise synchronization calculated with ISC has been used to identify time points where autistic and typically developing individuals’ brain activity diverges during movie viewing (Bolton et al., 2020). Features characterizing the time points of divergence can be extracted using a reverse-correlation approach. A similar data-driven, pairwise approach combined with emotion rating time series could be used to identify points in the movies when emotional processing converges or diverges (see, e.g., Bolt et al., 2018).

Finally, individual differences can be approached by identifying the regions which are differentially recruited across individuals (Chen et al., 2020). Inter-subject representational analysis (RSA; Finn et al., 2020) highlights the neural basis of individual differences in psychological and neural processes and their correspondence. For instance, Nummenmaa et al. (2014b) used RSA to compare the similarity of valence and arousal ratings to the similarity of brain activity between individuals. Jääskeläinen et al. (2016) used inter-subject synchronization of amusement rating time series to extract experiential similarity and identified regions where neural synchronization followed experienced between-subjects similarity. Finally, Smirnov et al. (2019) modeled experiential similarity time series and modeled regions where similarity increases when similarity of ratings increases.

## Conclusion

Naturalistic stimuli provide an important step forward in bringing ecologically valid stimuli to laboratory conditions. Future research will likely move toward even more naturalistic conditions, such as modeling emotions during real-life interaction and for multiple people. These approaches are now becoming possible due to rapid methodological developments.

However, the growing interest in naturalistic paradigms should always be guided by theoretical models (de Hamilton, 2013). Otherwise, we might end up with a pile of data, not knowing how to proceed. While the first naturalistic studies in affective neuroimaging were exploratory, proving that the paradigms work, we now have tools to employ in theoretical developments.

Affective neuroimaging studies using naturalistic stimuli have focused on a few features at the time, or at least on features from a single or a few components. Relatively few studies have combined emotion features across functional domains. The lack of multi-feature approaches might lead to too simplified explanations of complicated phenomena (Jolly and Chang, 2019). Focusing on modeling features across functional components would allow investigating the causal relationships between components. Together with predictive frameworks, the multi-feature approaches open numerous possibilities for integration across components. However, methodological developments are still needed to account for the complex interaction dynamics, such as feedback loops, between different component processes (e.g., Pessoa, 2018).

Naturalistic paradigms do not aim to replace the classic, controlled neuroimaging paradigms (Sonkusare et al., 2019). Due to their complexity and current limitations in understanding the statistical properties of different features in naturalistic conditions, naturalistic stimuli are not optimal for model development (see, e.g., Rust and Movshon, 2005). Controlled experiments are still needed for hypothesis testing and developing models, while naturalistic stimuli are best employed to test models in ecologically valid settings and to expand them to situations where context matters more.

Finally, a word of caution is warranted: the definition of a naturalistic experience in the context of emotions requires consideration. The term ‘naturalistic’ originates from vision studies, where video clips correspond to naturalistic viewing conditions in contrast to static images. However, films and stories do not necessarily cause real-life-like emotions (see, e.g., Parkinson and Manstead, 1993). The events unfold faster than in everyday life, and in real-life, people actively participate in the emotional exchange rather than merely observe events unfolding. Movie clips lasting for some minutes might not be able to elicit complex emotional experiences: while even brief scenes of explicit violence probably evoke strong experiences, more subtle emotions such as sympathy or admiration might require more contextual information provided by longer movie excerpts. Recent efforts have aimed to fill this gap by collecting data from entire movies rather than brief video clips (see, e.g., Lettieri et al., 2019; Aliko et al., 2020). Furthermore, two-person approaches might become important in the future (Hari and Kujala, 2009; Schilbach, 2010; Pfeiffer et al., 2013). However, the central question in second-person studies is the extraction of emotion-related features. Thus, naturalistic stimuli such as films and stories provide an essential source for candidate emotion features also for this research line. Finally, naturalistic neuroimaging paradigms typically focus on audio-visual stimulation. However, exteroceptive input from smell, touch, and taste might be crucial for evoking some emotional experiences, such as disgust or love. Neuroimaging settings are usually limited in the capability to evoke multisensory experiences, thus limiting the ecological validity of these settings.

## Author Contributions

HS conducted the literature review and wrote the manuscript.

## Conflict of Interest

The author declares that the research was conducted in the absence of any commercial or financial relationships that could be construed as a potential conflict of interest.

## References

[B1] Adolphs R. (2017). How should neuroscience study emotions? by distinguishing emotion states, concepts, and experiences. *Soc. Cogn. Affect. Neurosci.* 12 24–31. 10.1093/scan/nsw153 27798256 PMC5390692

[B2] Adolphs R. Nummenmaa L. Todorov A. Haxby J. V. (2016). Data-driven approaches in the investigation of social perception. *Philos. Trans. R. Soc. Lond. B Biol. Sci.* 371:20150367. 10.1098/rstb.2015.0367 27069045 PMC4843606

[B3] Aliko S. Huang J. Gheorghiu F. Meliss S. Skipper J. I. (2020). A naturalistic neuroimaging database for understanding the brain using ecological stimuli. *Sci. Data* 7 1–21. 10.1016/j.destud.2018.07.001 33051448 PMC7555491

[B4] Anderson D. J. Adolphs R. (2014). A framework for studying emotions across species. *Cell* 157 187–200. 10.1016/j.cell.2014.03.003 24679535 PMC4098837

[B5] Andric M. Goldin-Meadow S. Small S. L. Hasson U. (2016). Repeated movie viewings produce similar local activity patterns but different network configurations. *NeuroImage* 142 613–627. 10.1016/j.neuroimage.2016.07.061 27492251

[B6] Barrett L. F. (2017). The theory of constructed emotion: an active inference account of interoception and categorization. *Soc. Cogn. Affect. Neurosci.* 12:1833. 10.1093/scan/nsx060 28472391 PMC5691871

[B7] Barrett L. F. Adolphs R. Marsella S. Martinez A. M. Pollak S. D. (2019). Emotional expressions reconsidered: challenges to inferring emotion from human facial movements. *Psychol. Sci. Public Interest* 20 1–68. 10.1177/1529100619832930 31313636 PMC6640856

[B8] Barrett L. F. Mesquita B. Ochsner K. N. Gross J. J. (2007). The experience of emotion. *Annu. Rev. Psychol.* 58 373–403. 17002554 10.1146/annurev.psych.58.110405.085709 PMC1934613

[B9] Bartels A. Zeki S. (2005). Brain dynamics during natural viewing conditions—a new guide for mapping connectivity in vivo. *Neuroimage* 24 339–349. 10.1016/j.neuroimage.2004.08.044 15627577

[B10] Baumeister R. F. Zhang L. Vohs K. D. (2004). Gossip as cultural learning. *Rev. Gen. Psychol.* 8 111–121. 10.1037/1089-2680.8.2.111

[B11] Bolt T. Nomi J. S. Vij S. G. Chang C. Uddin L. Q. (2018). Inter-subject phase synchronization for exploratory analysis of task-fMRI. *NeuroImage* 176 477–488. 10.1016/j.neuroimage.2018.04.015 29654878

[B12] Bolton T. A. W. Freitas L. G. A. Jochaut D. Giraud A.-L. Van De Ville D. (2020). Neural responses in autism during movie watching: Inter-individual response variability co-varies with symptomatology. *NeuroImage* 216:116571. 10.1016/j.neuroimage.2020.116571 31987996

[B13] Bolton T. A. W. Jochaut D. Giraud A.-L. Van De Ville D. (2018). Brain dynamics in ASD during movie-watching show idiosyncratic functional integration and segregation. *Hum. Brain Mapp.* 39 2391–2404. 10.1002/hbm.24009 29504186 PMC5969252

[B14] Boltz M. G. (2001). Musical soundtracks as a schematic influence on the cognitive processing of filmed events. *Music Percept.* 18 427–454. 10.1525/mp.2001.18.4.427 33021500

[B15] Borchardt V. Surova G. van der Meer J. Bola M. Frommer J. Leutritz A. L. (2018). Exposure to attachment narratives dynamically modulates cortical arousal during the resting state in the listener. *Brain Behav.* 8:e01007. 10.1002/brb3.1007 29877060 PMC6043700

[B16] Bordwell D. Thompson K. (2008). *Film Art: An Introduction.* New York, NY: McGraw Hill.

[B17] Borja Jimenez K. C. Abdelgabar A. R. De Angelis L. McKay L. S. Keysers C. Gazzola V. (2020). Changes in brain activity following the voluntary control of empathy. *NeuroImage* 216:116529. 10.1016/j.neuroimage.2020.116529 31931155

[B18] Bradley M. M. Lang P. J. (2007a). *Affective Norms for English Text (ANET): Affective Ratings of Text and Instruction Manual.* Tech. Rep. No. D-1. Gainesville, FL: University of Florida.

[B19] Bradley M. M. Lang P. J. (2007b). *The International Affective Digitized Sounds (IADS-2): Affective Ratings of Sounds and Instruction Manual.* Tech. Rep. B-3. Gainesville, FL: University of Florida.

[B20] Brandman T. Malach R. Simony E. (2021). The surprising role of the default mode network in naturalistic perception. *Commun. Biol.* 4 1–9. 33469113 10.1038/s42003-020-01602-z PMC7815915

[B21] Carroll N. Seeley W. P. (2013). “Cognitivism, psychology, and neuroscience: movies as attentional engines,” in *Psychocinematics: Exploring Cognition at the Movies*, ed. Shimamura A. P. (Oxford: Oxford University Press), 53–75. 10.1093/acprof:oso/9780199862139.003.0003

[B22] Chan H.-Y. Smidts A. Schoots V. C. Dietvorst R. C. Boksem M. A. S. (2019). Neural similarity at temporal lobe and cerebellum predicts out-of-sample preference and recall for video stimuli. *NeuroImage* 197 391–401. 10.1016/j.neuroimage.2019.04.076 31051296

[B23] Chan H.-Y. Smidts A. Schoots V. C. Sanfey A. G. Boksem M. A. S. (2020). Decoding dynamic affective responses to naturalistic videos with shared neural patterns. *NeuroImage* 216:116618. 10.1016/j.neuroimage.2020.116618 32036021

[B24] Chang C. Manning J. Baldassano C. de la Vega A. Fleetwood G. Finn E. (2020). *Neuroimaging analysis methods for naturalistic data. Version 1.0.*

[B25] Chang C. Thomason M. E. Glover G. H. (2008). Mapping and correction of vascular hemodynamic latency in the BOLD signal. *Neuroimage* 43 90–102. 10.1016/j.neuroimage.2008.06.030 18656545 PMC2587338

[B26] Chang L. J. Jolly E. Cheong J. H. Rapuano K. M. Greenstein N. Chen P. H. A. (2021). Endogenous variation in ventromedial prefrontal cortex state dynamics during naturalistic viewing reflects affective experience. *Sci. Adv.* 7:eabf7129. 10.1126/sciadv.abf7129 33893106 PMC8064646

[B27] Chapin H. Jantzen K. Kelso J. A. S. Steinberg F. Large E. (2010). Dynamic emotional and neural responses to music depend on performance expression and listener experience. *PLoS One* 5:e13812. 10.1371/journal.pone.0013812 21179549 PMC3002933

[B28] Chen P.-H. A. Jolly E. Cheong J. H. Chang L. J. (2020). Intersubject representational similarity analysis reveals individual variations in affective experience when watching erotic movies. *NeuroImage* 216:116851. 10.1016/j.neuroimage.2020.116851 32294538 PMC7955800

[B29] Chikazoe J. Lee D. H. Kriegeskorte N. Anderson A. K. (2014). Population coding of affect across stimuli, modalities and individuals. *Nat. Neurosci.* 17 1114–1122. 10.1038/nn.3749 24952643 PMC4317366

[B30] Coutinho E. Cangelosi A. (2011). Musical emotions: predicting second-by-second subjective feelings of emotion from low-level psychoacoustic features and physiological measurements. *Emotion* 11 921–937. 10.1037/a0024700 21859207

[B31] Cowen A. S. Keltner D. (2017). Self-report captures 27 distinct categories of emotion bridged by continuous gradients. *Proc. Natl. Acad. Sci. U.S.A.* 114 E7900–E7909. 28874542 10.1073/pnas.1702247114 PMC5617253

[B32] Craig A. D. (2002). How do you feel? Interoception: the sense of the physiological condition of the body. *Nat. Rev. Neurosci.* 3 655–666. 10.1038/nrn894 12154366

[B33] Critchley H. D. Wiens S. Rotshtein P. Ohman A. Dolan R. J. (2004). Neural systems supporting interoceptive awareness. *Nat. Neurosci.* 7 189–195. 10.1038/nn1176 14730305

[B34] Damasio A. Carvalho G. B. (2013). The nature of feelings: evolutionary and neurobiological origins. *Nat. Rev. Neurosci.* 14 143–152. 10.1038/nrn3403 23329161

[B35] Damasio A. R. Grabowski T. J. Bechara A. Damasio H. Ponto L. L. Parvizi J. (2000). Subcortical and cortical brain activity during the feeling of self-generated emotions. *Nat. Neurosci.* 3 1049–1056. 10.1038/79871 11017179

[B36] De Gelder B. Bertelson P. (2003). Multisensory integration, perception and ecological validity. *Trends Cogn. Sci.* 7 460–467. 10.1016/j.tics.2003.08.014 14550494

[B37] de Hamilton A. F. C. (2013). Second person neuroscience needs theories as well as methods. *Behav. Brain Sci.* 36 425–426. 10.1017/s0140525x1200194x 23883754

[B38] de Heer W. A. Huth A. G. Griffiths T. L. Gallant J. L. Theunissen F. E. (2017). The hierarchical cortical organization of human speech processing. *J. Neurosci.* 37 6539–6557. 10.1523/jneurosci.3267-16.2017 28588065 PMC5511884

[B39] Devillers L. Cowie R. Martin J.-C. Douglas-Cowie E. Abrilian S. McRorie M. (2006). “Real life emotions in French and English TV video clips: an integrated annotation protocol combining continuous and discrete approaches,” in *Proceedings of the Fifth International Conference on Language Resources and Evaluation (LREC’06)*, Genoa, 1105–1110.

[B40] Dunbar R. I. M. (2004). Gossip in evolutionary perspective. *Rev. Gen. Psychol.* 8 100–110. 10.1037/1089-2680.8.2.100

[B41] DuPre E. Hanke M. Poline J.-B. (2020). Nature abhors a paywall: how open science can realize the potential of naturalistic stimuli. *NeuroImage* 216:116330. 10.1016/j.neuroimage.2019.116330 31704292 PMC7198323

[B42] Eerola T. Toiviainen P. (2004). *MIDI Toolbox: MATLAB Tools for Music Research.* Kopijyvä: University of Jyväskylä.

[B43] Eisenbarth H. Chang L. J. Wager T. D. (2016). Multivariate brain prediction of heart rate and skin conductance responses to social threat. *J. Neurosci.* 36 11987–11998. 10.1523/jneurosci.3672-15.2016 27881783 PMC5125248

[B44] Ekman P. (1992). An argument for basic emotions. *Cogn. Emot.* 6 169–200. 10.1080/02699939208411068

[B45] Ekman P. Oster H. (1979). Facial expressions of emotion. *Annu. Rev. Psychol.* 30 527–554.

[B46] Eldar E. Ganor O. Admon R. Bleich A. Hendler T. (2007). Feeling the real world: limbic response to music depends on related content. *Cereb. Cortex* 17 2828–2840. 10.1093/cercor/bhm011 17395609

[B47] Ellsworth P. C. Scherer K. R. (2003). *Appraisal Processes in Emotion.* Oxford: Oxford University Press.

[B48] Ethofer T. Van De Ville D. Scherer K. Vuilleumier P. (2009). Decoding of emotional information in voice-sensitive cortices. *Curr. Biol.* 19 1028–1033. 10.1016/j.cub.2009.04.054 19446457

[B49] Etzel J. A. Johnsen E. L. Dickerson J. Tranel D. Adolphs R. (2006). Cardiovascular and respiratory responses during musical mood induction. *Int. J. Psychophysiol.* 61 57–69. 10.1016/j.ijpsycho.2005.10.025 16460823

[B50] Finn E. S. Glerean E. Khojandi A. Y. Nielson D. Molfese P. J. Handwerker D. A. (2020). Idiosynchrony: from shared responses to individual differences during naturalistic neuroimaging. *NeuroImage* 215:116828. 10.1016/j.neuroimage.2020.116828 32276065 PMC7298885

[B51] Frijda N. H. (2009). Emotions, individual differences and time course: reflections. *Cogn. Emot.* 23 1444–1461. 10.1080/02699930903093276

[B52] Frijda N. H. Mesquita B. Sonnemans J. Van Goozen S. (1991). *The Duration of Affective Phenomena or Emotions, Sentiments and Passions.* Hoboken, NJ: Wiely.

[B53] Friston K. J. (2011). Functional and effective connectivity: a review. *Brain Connect.* 1 13–36. 10.1089/brain.2011.0008 22432952

[B54] Friston K. J. Harrison L. Penny W. (2003). Dynamic causal modelling. *Neuroimage* 19 1273–1302. 10.1016/s1053-8119(03)00202-7 12948688

[B55] Genevsky A. Yoon C. Knutson B. (2017). When brain beats behavior: neuroforecasting crowdfunding outcomes. *J. Neurosci.* 37 8625–8634. 10.1523/jneurosci.1633-16.2017 28821681 PMC5588458

[B56] Glerean E. Salmi J. Lahnakoski J. M. Jääskeläinen I. P. Sams M. (2012). Functional magnetic resonance imaging phase synchronization as a measure of dynamic functional connectivity. *Brain Connect.* 2 91–101. 10.1089/brain.2011.0068 22559794 PMC3624768

[B57] Goldberg H. Preminger S. Malach R. (2014). The emotion–action link? Naturalistic emotional stimuli preferentially activate the human dorsal visual stream. *Neuroimage* 84 254–264. 10.1016/j.neuroimage.2013.08.032 23994457

[B58] Goldin P. R. Hutcherson C. A. Ochsner K. N. Glover G. H. Gabrieli J. D. Gross J. J. (2005). The neural bases of amusement and sadness: a comparison of block contrast and subject-specific emotion intensity regression approaches. *Neuroimage* 27 26–36. 10.1016/j.neuroimage.2005.03.018 15890534

[B59] Golland Y. Keissar K. Levit-Binnun N. (2014). Studying the dynamics of autonomic activity during emotional experience. *Psychophysiology* 51 1101–1111. 10.1111/psyp.12261 25039415

[B60] Golland Y. Levit-Binnun N. Hendler T. Lerner Y. (2017). Neural dynamics underlying emotional transmissions between individuals. *Soc. Cogn. Affect. Neurosci.* 12 1249–1260. 10.1093/scan/nsx049 28575520 PMC5597885

[B61] Gomez P. Danuser B. (2007). Relationships between musical structure and psychophysiological measures of emotion. *Emotion* 7:377. 10.1037/1528-3542.7.2.377 17516815

[B62] Gross J. J. (2015). Emotion regulation: current status and future prospects. *Psychol. Inq.* 26 1–26. 10.1080/1047840x.2014.940781

[B63] Gross J. J. Levenson R. W. (1995). Emotion elicitation using films. *Cogn. Emot.* 9 87–108. 10.1080/02699939508408966

[B64] Gruskin D. C. Rosenberg M. D. Holmes A. J. (2020). Relationships between depressive symptoms and brain responses during emotional movie viewing emerge in adolescence. *NeuroImage* 216:116217. 10.1016/j.neuroimage.2019.116217 31628982 PMC7958984

[B65] Hari R. Kujala M. V. (2009). Brain basis of human social interaction: from concepts to brain imaging. *Physiol. Rev.* 89 453–479. 10.1152/physrev.00041.2007 19342612

[B66] Hasson U. Landesman O. Knappmeyer B. Vallines I. Rubin N. Heeger D. J. (2008). Neurocinematics: the neuroscience of film. *Projections* 2 1–26. 10.3167/proj.2008.020102

[B67] Hasson U. Nir Y. Levy I. Fuhrmann G. Malach R. (2004). Intersubject synchronization of cortical activity during natural vision. *Science* 303 1634–1640. 10.1126/science.1089506 15016991

[B68] Horikawa T. Cowen A. S. Keltner D. Kamitani Y. (2020). The neural representation of visually evoked emotion is high-dimensional, categorical, and distributed across transmodal brain regions. *iScience* 23:101060. 10.1016/j.isci.2020.101060 32353765 PMC7191651

[B69] Hsu C.-T. Jacobs A. M. Citron F. M. M. Conrad M. (2015). The emotion potential of words and passages in reading Harry Potter–an fMRI study. *Brain Lang.* 142 96–114. 10.1016/j.bandl.2015.01.011 25681681

[B70] Hudson M. Seppälä K. Putkinen V. Sun L. Glerean E. Karjalainen T. (2020). Dissociable neural systems for unconditioned acute and sustained fear. *NeuroImage* 216:116522. 10.1016/j.neuroimage.2020.116522 31926280

[B71] Hutcherson C. A. Goldin P. R. Ochsner K. N. Gabrieli J. D. Feldman Barrett L. Gross J. J. (2005). Attention and emotion: does rating emotion alter neural responses to amusing and sad films? *NeuroImage* 27 656–668. 10.1016/j.neuroimage.2005.04.028 15946863

[B72] Huth A. G. Lee T. Nishimoto S. Bilenko N. Y. Vu A. T. Gallant J. L. (2016). Decoding the semantic content of natural movies from human brain activity. *Front. Syst. Neurosci.* 10:81. 10.3389/fnsys.2016.00081 27781035 PMC5057448

[B73] Huth A. G. Nishimoto S. Vu A. T. Gallant J. L. (2012). A continuous semantic space describes the representation of thousands of object and action categories across the human brain. *Neuron* 76 1210–1224. 10.1016/j.neuron.2012.10.014 23259955 PMC3556488

[B74] Iidaka T. (2017). Humor appreciation involves parametric and synchronized activity in the medial prefrontal cortex and hippocampus. *Cereb. Cortex* 27 5579–5591. 27756763 10.1093/cercor/bhw325

[B75] Imbir K. K. (2016). Affective norms for 718 polish short texts (ANPST): dataset with affective ratings for valence, arousal, dominance, origin, subjective significance and source dimensions. *Front. Psychol.* 7:855. 10.3389/fpsyg.2017.00855 27458420 PMC4930931

[B76] Jääskeläinen I. P. Klucharev V. Panidi K. Shestakova A. N. (2020). Neural processing of narratives: from individual processing to viral propagation. *Front. Hum. Neurosci.* 14:253. 10.3389/fnhum.2020.00253 32676019 PMC7333591

[B77] Jääskeläinen I. P. Koskentalo K. Balk M. H. Autti T. Kauramäki J. Pomren C. (2008). Inter-subject synchronization of prefrontal cortex hemodynamic activity during natural viewing. *Open Neuroimaging J.* 2 14–19. 10.2174/1874440000802010014 19018313 PMC2577941

[B78] Jääskeläinen I. P. Pajula J. Tohka J. Lee H. J. Kuo W. J. Lin F. H. (2016). Brain hemodynamic activity during viewing and re-viewing of comedy movies explained by experienced humor. *Sci. Rep.* 6 1–14. 10.2307/j.ctvh8qxrv.6 27323928 PMC4914983

[B79] Jääskeläinen I. P. Sams M. Glerean E. Ahveninen J. (2021). Movies and narratives as naturalistic stimuli in neuroimaging. *NeuroImage* 224:117445. 10.1016/j.neuroimage.2020.117445 33059053 PMC7805386

[B80] Jacob Y. Gilam G. Lin T. Raz G. Hendler T. (2018). Anger modulates influence hierarchies within and between emotional reactivity and regulation networks. *Front. Behav. Neurosci.* 12:60. 10.3389/fnbeh.2018.00060 29681803 PMC5897670

[B81] Jacob Y. Winetraub Y. Raz G. Ben-Simon E. Okon-Singer H. Rosenberg-Katz K. (2016). Dependency network analysis (d ep na) reveals context related influence of brain network nodes. *Sci. Rep.* 6 1–19. 27271458 10.1038/srep27444 PMC4895213

[B82] Jacobs A. M. (2015). Neurocognitive poetics: methods and models for investigating the neuronal and cognitive-affective bases of literature reception. *Front. Hum. Neurosci.* 9:186. 10.3389/fnhum.2015.00186 25932010 PMC4399337

[B83] Jang C. Knight E. Q. Pae C. Park B. Yoon S. A. Park H. J. (2017). Individuality manifests in the dynamic reconfiguration of large-scale brain networks during movie viewing. *Sci. Rep.* 7 1–14. 28112247 10.1038/srep41414 PMC5256084

[B84] Jolly E. Chang L. J. (2019). The flatland fallacy: moving beyond low-dimensional thinking. *Top. Cogn. Sci.* 11 433–454. 10.1111/tops.12404 30576066 PMC6519046

[B85] Jolly E. Chang L. J. (2021). Multivariate spatial feature selection in fMRI. *Soc. Cogn. Affect. Neurosci.* [Epub ahead of print]. 10.1093/scan/nsab010 PMC8343556 33501987

[B86] Juslin P. N. (2013). From everyday emotions to aesthetic emotions: towards a unified theory of musical emotions. *Phys. Life Rev.* 10 235–266. 10.1016/j.plrev.2013.05.008 23769678

[B87] Kauttonen J. Hlushchuk Y. Tikka P. (2015). Optimizing methods for linking cinematic features to fMRI data. *Neuroimage* 110 136–148. 10.1016/j.neuroimage.2015.01.063 25662868

[B88] Kay K. N. Gallant J. L. (2009). I can see what you see. *Nat. Neurosci.* 12 245–245. 19238184 10.1038/nn0309-245

[B89] Khalsa S. S. Adolphs R. Cameron O. G. Critchley H. D. Davenport P. W. Feinstein J. S. (2018). Interoception and mental health: a roadmap. *Biol. Psychiatry* 3 501–513. 10.1016/j.bpsc.2017.12.004 PMC6054486 29884281

[B90] Kober H. Barrett L. F. Joseph J. Bliss-Moreau E. Lindquist K. Wager T. D. (2008). Functional grouping and cortical–subcortical interactions in emotion: a meta-analysis of neuroimaging studies. *NeuroImage* 42 998–1031. 10.1016/j.neuroimage.2008.03.059 18579414 PMC2752702

[B91] Koelsch S. (2010). Towards a neural basis of music-evoked emotions. *Trends Cogn. Sci.* 14 131–137. 10.1016/j.tics.2010.01.002 20153242

[B92] Koelsch S. Fritz T. V Cramon D. Y. Müller K. Friederici A. D. (2006). Investigating emotion with music: an fMRI study. *Hum. Brain Mapp.* 27 239–250. 10.1002/hbm.20180 16078183 PMC6871371

[B93] Koide-Majima N. Nakai T. Nishimoto S. (2020). Distinct dimensions of emotion in the human brain and their representation on the cortical surface. *NeuroImage* 222:117258. 10.1016/j.neuroimage.2020.117258 32798681

[B94] Kragel P. A. LaBar K. S. (2015). Multivariate neural biomarkers of emotional states are categorically distinct. *Soc. Cogn. Affect. Neurosci.* 10 1437–1448. 10.1093/scan/nsv032 25813790 PMC4631142

[B95] Kragel P. A. LaBar K. S. (2016). Decoding the nature of emotion in the brain. *Trends Cogn. Sci.* 20 444–455. 10.1016/j.tics.2016.03.011 27133227 PMC4875847

[B96] Kragel P. A. Reddan M. C. LaBar K. S. Wager T. D. (2019). Emotion schemas are embedded in the human visual system. *Sci. Adv.* 5:eaaw4358. 10.1126/sciadv.aaw4358 31355334 PMC6656543

[B97] Labs A. Reich T. Schulenburg H. Boennen M. Mareike G. Golz M. (2015). Portrayed emotions in the movie “Forrest Gump.”. *F1000Research* 4:92. 10.12688/f1000research.6230.1 25977755 PMC4416536

[B98] Lahnakoski J. M. Glerean E. Jääskeläinen I. P. Hyönä J. Hari R. Sams M. (2014). Synchronous brain activity across individuals underlies shared psychological perspectives. *NeuroImage* 100 316–324. 10.1016/j.neuroimage.2014.06.022 24936687 PMC4153812

[B99] Lahnakoski J. M. Glerean E. Salmi J. Jääskeläinen I. P. Sams M. Hari R. (2012). Naturalistic FMRI mapping reveals superior temporal sulcus as the hub for the distributed brain network for social perception. *Front. Hum. Neurosci.* 6:233. 10.3389/fnhum.2012.00233 22905026 PMC3417167

[B100] Lang P. Bradley M. M. (2007). The International Affective Picture System (IAPS) in the study of emotion and attention. *Handb. Emot. Elicitation Assess.* 29 70–73.

[B101] Lartillot O. Toiviainen P. (2007). A Matlab toolbox for musical feature extraction from audio. *Int. Conf. Digital Audio Effects* 237:244.

[B102] LeDoux J. (2012). Rethinking the emotional brain. *Neuron* 73 653–676. 10.1016/j.neuron.2012.02.004 22365542 PMC3625946

[B103] LeDoux J. E. Hofmann S. G. (2018). The subjective experience of emotion: a fearful view. *Curr. Opin. Behav. Sci.* 19 67–72. 10.1016/j.cobeha.2017.09.011 PMC11444666 39355512

[B104] Lehne M. Engel P. Rohrmeier M. Menninghaus W. Jacobs A. M. Koelsch S. (2015). Reading a suspenseful literary text activates brain areas related to social cognition and predictive inference. *PLoS One* 10:e0124550. 10.1371/journal.pone.0124550 25946306 PMC4422438

[B105] Lettieri G. Handjaras G. Ricciardi E. Leo A. Papale P. Betta M. (2019). Emotionotopy in the human right temporo-parietal cortex. *Nat. Commun.* 10:5568. 10.1038/s41467-019-13599-z PMC6895053 31804504

[B106] Lieberman M. D. Eisenberger N. I. Crockett M. J. Tom S. M. Pfeifer J. H. Way B. M. (2007). Putting feelings into words. *Psychol. Sci.* 18 421–428. 10.1111/j.1467-9280.2007.01916.x 17576282

[B107] Lindquist K. A. Satpute A. B. Gendron M. (2015). Does language do more than communicate emotion? *Curr. Direct. Psychol. Sci.* 24 99–108. 10.1177/0963721414553440 25983400 PMC4428906

[B108] Malandrakis N. Potamianos A. Evangelopoulos G. Zlatintsi A. (2011). “A supervised approach to movie emotion tracking,” in *Proceedings of the 2011 IEEE International Conference on Acoustics, Speech and Signal Processing (ICASSP)*, Prague, 2376–2379.

[B109] Man V. Nohlen H. U. Melo H. Cunningham W. A. (2017). Hierarchical brain systems support multiple representations of valence and mixed affect. *Emot. Rev.* 9 124–132. 10.1177/1754073916667237

[B110] Mar R. A. (2011). The neural bases of social cognition and story comprehension. *Annu. Rev. Psychol.* 62 103–134. 10.1146/annurev-psych-120709-145406 21126178

[B111] Marchewka A. Zurawski Ł Jednoróg K. Grabowska A. (2014). The Nencki Affective Picture System (NAPS): introduction to a novel, standardized, wide-range, high-quality, realistic picture database. *Behav. Res. Methods* 46 596–610. 10.3758/s13428-013-0379-1 23996831 PMC4030128

[B112] Mason R. A. Just M. A. (2009). The role of the theory-of-mind cortical network in the comprehension of narratives. *Lang. Linguist. Compass* 3 157–174. 10.1111/j.1749-818x.2008.00122.x 19809575 PMC2756681

[B113] Mauss I. B. Robinson M. D. (2009). Measures of emotion: a review. *Cogn. Emot.* 23 209–237. 10.1080/02699930802204677 19809584 PMC2756702

[B114] McNamara Q. De La Vega A. Yarkoni T. (2017). “Developing a comprehensive framework for multimodal feature extraction,” in *Proceedings of the 23rd ACM SIGKDD International Conference on Knowledge Discovery and Data Mining*, New York, NY, 1567–1574.

[B115] Menon V. (2015). “Salience Network,” in *Brain Mapping: An Encyclopedic Reference*, Vol. 2 ed. Toga A. W. (Cambridge, MA: Academic Press), 597–611.

[B116] Metallinou A. Narayanan S. (2013). “Annotation and processing of continuous emotional attributes: challenges and opportunities,” in *Proceedings of the 2013 10th IEEE International Conference and Workshops on Automatic Face and Gesture Recognition (FG)*, Shanghai, 1–8. 10.13188/2469-4185.1000030

[B117] Miskovic V. Anderson A. K. (2018). Modality general and modality specific coding of hedonic valence. *Curr. Opin. Behav. Sci.* 19 91–97. 10.1016/j.cobeha.2017.12.012 29967806 PMC6024250

[B118] Mobbs D. Adolphs R. Fanselow M. S. Barrett L. F. LeDoux J. E. Ressler K. (2019). Viewpoints: approaches to defining and investigating fear. *Nat. Neurosci.* 22 1205–1216. 10.1038/s41593-019-0456-6 31332374 PMC6943931

[B119] Moors A. (2009). Theories of emotion causation: a review. *Cogn. Emot.* 23 625–662. 10.1080/02699930802645739

[B120] Moran J. M. Wig G. S. Adams R. B. Jr. Janata P. Kelley W. M. (2004). Neural correlates of humor detection and appreciation. *NeuroImage* 21 1055–1060. 10.1016/j.neuroimage.2003.10.017 15006673

[B121] Mulligan K. Scherer K. R. (2012). Toward a working definition of emotion. *Emot. Rev.* 4 345–357. 10.1177/1754073912445818

[B122] Naci L. Cusack R. Anello M. Owen A. M. (2014). A common neural code for similar conscious experiences in different individuals. *Proc. Natl. Acad. Sci. U.S.A.* 111 14277–14282. 10.1073/pnas.1407007111 25225384 PMC4191782

[B123] Nagel F. Kopiez R. Grewe O. Altenmüller E. (2007). EMuJoy: software for continuous measurement of perceived emotions in music. *Behav. Res. Methods* 39 283–290. 10.3758/bf03193159 17695356

[B124] Nanni M. Martínez-Soto J. Gonzalez-Santos L. Barrios F. A. (2018). Neural correlates of the natural observation of an emotionally loaded video. *PLoS One* 13:e0198731. 10.1371/journal.pone.0198731 29883494 PMC5993250

[B125] Naselaris T. Kay K. N. Nishimoto S. Gallant J. L. (2011). Encoding and decoding in fMRI. *Neuroimage* 56 400–410. 10.1016/j.neuroimage.2010.07.073 20691790 PMC3037423

[B126] Nastase S. A. Gazzola V. Hasson U. Keysers C. (2019). Measuring shared responses across subjects using intersubject correlation. *Soc. Cogn. Affect. Neurosci.* 14 667–685. 31099394 10.1093/scan/nsz037 PMC6688448

[B127] Nastase S. A. Goldstein A. Hasson U. (2020). Keep it real: rethinking the primacy of experimental control in cognitive neuroscience. *NeuroImage* 222:117254. 10.1016/j.neuroimage.2020.117254 32800992 PMC7789034

[B128] Nguyen V. T. Breakspear M. Hu X. Guo C. C. (2016). The integration of the internal and external milieu in the insula during dynamic emotional experiences. *NeuroImage* 124 455–463. 10.1016/j.neuroimage.2015.08.078 26375211

[B129] Nisbett R. E. Wilson T. D. (1977). Telling more than we can know: verbal reports on mental processes. *Psychol. Rev.* 84 231–259. 10.1037/0033-295x.84.3.231

[B130] Norman K. A. Polyn S. M. Detre G. J. Haxby J. V. (2006). Beyond mind-reading: multi-voxel pattern analysis of fMRI data. *Trends Cogn. Sci.* 10 424–430. 10.1016/j.tics.2006.07.005 16899397

[B131] Nummenmaa L. Glerean E. Hari R. Hietanen J. K. (2014a). Bodily maps of emotions. *Proc. Natl. Acad. Sci. U.S.A.* 111 646–651. 24379370 10.1073/pnas.1321664111 PMC3896150

[B132] Nummenmaa L. Glerean E. Viinikainen M. Jääskeläinen I. P. Hari R. Sams M. (2012). Emotions promote social interaction by synchronizing brain activity across individuals. *Proc. Natl. Acad. Sci. U.S.A.* 109 9599–9604. 10.1073/pnas.1206095109 22623534 PMC3386135

[B133] Nummenmaa L. Lahnakoski J. M. Glerean E. (2018). Sharing the social world via intersubject neural synchronisation. *Curr. Opin. Psychol.* 24 7–14. 10.1016/j.copsyc.2018.02.021 29550395

[B134] Nummenmaa L. Saarimäki H. (2019). Emotions as discrete patterns of systemic activity. *Neurosci. Lett.* 693 3–8. 10.1016/j.neulet.2017.07.012 28705730

[B135] Nummenmaa L. Saarimäki H. Glerean E. Gotsopoulos A. Jääskeläinen I. P. Hari R. (2014b). Emotional speech synchronizes brains across listeners and engages large-scale dynamic brain networks. *NeuroImage* 102 (Pt 2), 498–509. 10.1016/j.neuroimage.2014.07.063 25128711 PMC4229500

[B136] Nummenmaa L. Smirnov D. Lahnakoski J. M. Glerean E. Jääskeläinen I. P. Sams M. (2014c). Mental action simulation synchronizes action-observation circuits across individuals. *J. Neurosci.* 34 748–757. 10.1523/jneurosci.0352-13.2014 24431433 PMC3891955

[B137] Okon-Singer H. Lichtenstein-Vidne L. Cohen N. (2013). Dynamic modulation of emotional processing. *Biol. Psychol.* 92 480–491. 10.1016/j.biopsycho.2012.05.010 22676964

[B138] Panksepp J. (1992). A critical role for “affective neuroscience” in resolving what is basic about basic emotions. *Psychol. Rev.* 99 554–560. 10.1037/0033-295x.99.3.554 1502276

[B139] Parkinson B. Manstead A. S. R. (1993). Making sense of emotion in stories and social life. *Cogn. Emot.* 7 295–323. 10.1080/02699939308409191

[B140] Pehrs C. Deserno L. Bakels J.-H. Schlochtermeier L. H. Kappelhoff H. Jacobs A. M. (2013). How music alters a kiss: superior temporal gyrus controls fusiform–amygdalar effective connectivity. *Soc. Cogn. Affect. Neurosci.* 9 1770–1778. 10.1093/scan/nst169 24298171 PMC4221214

[B141] Pessoa L. (2018). Emotion and the interactive brain: insights from comparative neuroanatomy and complex systems. *Emot. Rev.* 10 204–216. 10.1177/1754073918765675 31537985 PMC6752744

[B142] Pessoa L. Adolphs R. (2010). Emotion processing and the amygdala: from a ‘low road’ to ‘many roads’ of evaluating biological significance. *Nat. Rev. Neurosci.* 11 773–782. 10.1038/nrn2920 20959860 PMC3025529

[B143] Petitmengin C. Remillieux A. Cahour B. Carter-Thomas S. (2013). A gap in Nisbett and Wilson’s findings? A first-person access to our cognitive processes. *Conscious. Cogn.* 22 654–669. 10.1016/j.concog.2013.02.004 23719334

[B144] Pfeiffer C. Hollenstein N. Zhang C. Langer N. (2020). Neural dynamics of sentiment processing during naturalistic sentence reading. *NeuroImage* 218:116934. 10.1016/j.neuroimage.2020.116934 32416227

[B145] Pfeiffer U. J. Timmermans B. Vogeley K. Frith C. D. Schilbach L. (2013). Towards a neuroscience of social interaction. *Front. Hum. Neurosci.* 7:22. 10.3389/fnhum.2013.00022 23378836 PMC3561599

[B146] Pinheiro A. P. Dias M. Pedrosa J. Soares A. P. (2017). Minho affective sentences (MAS): probing the roles of sex, mood, and empathy in affective ratings of verbal stimuli. *Behav. Res. Methods* 49 698–716. 10.3758/s13428-016-0726-0 27004484

[B147] Plantinga C. (1999). “The scene of empathy and the human face on film,” in *Passionate Views: Film, Cognition, and Emotion*, eds Plantinga C. R. Smith G. M. (Netherlands: BRILL), 239–255.

[B148] Plantinga C. (2013). “The affective power of movies,” in *Psychocinematics: Exploring cognition at the movies*, ed. Shimamura A. P. (Oxford: Oxford University Press), 94–111. 10.1093/acprof:oso/9780199862139.003.0005

[B149] Pujol J. Blanco-Hinojo L. Coronas R. Esteba-Castillo S. Rigla M. Martínez-Vilavella G. (2018). Mapping the sequence of brain events in response to disgusting food. *Hum. Brain Mapp.* 39 369–380. 10.1002/hbm.23848 29024175 PMC6866415

[B150] Raz G. Jacob Y. Gonen T. Winetraub Y. Flash T. Soreq E. (2014). Cry for her or cry with her: context-dependent dissociation of two modes of cinematic empathy reflected in network cohesion dynamics. *Soc. Cogn. Affect. Neurosci.* 9 30–38. 10.1093/scan/nst052 23615766 PMC3871736

[B151] Raz G. Touroutoglou A. Wilson-Mendenhall C. Gilam G. Lin T. Gonen T. (2016). Functional connectivity dynamics during film viewing reveal common networks for different emotional experiences. *Cogn. Affect. Behav. Neurosci.* 16 709–723. 10.3758/s13415-016-0425-4 27142636

[B152] Raz G. Winetraub Y. Jacob Y. Kinreich S. Maron-Katz A. Shaham G. (2012). Portraying emotions at their unfolding: a multilayered approach for probing dynamics of neural networks. *NeuroImage* 60 1448–1461. 10.1016/j.neuroimage.2011.12.084 22285693

[B153] Redondo J. Fraga I. Padrón I. Comesaña M. (2007). The Spanish adaptation of ANEW (affective norms for English words). *Behav. Res. Methods* 39 600–605. 10.3758/bf03193031 17958173

[B154] Russell J. A. (1980). A circumplex model of affect. *J. Pers. Soc. Psychol.* 9 1161–1178. 10.1037/h0077714

[B155] Rust N. C. Movshon J. A. (2005). In praise of artifice. *Nat. Neurosci.* 8 1647–1650. 10.1038/nn1606 16306892

[B156] Saarimäki H. Ejtehadian L. F. Glerean E. Jääskeläinen I. P. Vuilleumier P. Sams M. (2018). Distributed affective space represents multiple emotion categories across the human brain. *Soc. Cogn. Affect. Neurosci.* 13 471–482. 10.1093/scan/nsy018 29618125 PMC6007366

[B157] Saarimäki H. Gotsopoulos A. Jääskeläinen I. P. Lampinen J. Vuilleumier P. Hari R. (2016). Discrete neural signatures of basic emotions. *Cereb. Cortex* 26 2563–2573. 10.1093/cercor/bhv086 25924952

[B158] Sachs M. E. Damasio A. Habibi A. (2015). The pleasures of sad music: a systematic review. *Front. Hum. Neurosci.* 9:404. 10.3389/fnhum.2015.00404 26257625 PMC4513245

[B159] Sachs M. E. Habibi A. Damasio A. Kaplan J. T. (2020). Dynamic intersubject neural synchronization reflects affective responses to sad music. *NeuroImage* 218:116512. 10.1016/j.neuroimage.2019.116512 31901418

[B160] Salmi J. Glerean E. Jääskeläinen I. P. Lahnakoski J. M. Kettunen J. Lampinen J. (2014). Posterior parietal cortex activity reflects the significance of others’ actions during natural viewing. *Hum. Brain Mapp.* 35 4767–4776. 10.1002/hbm.22510 24706557 PMC6869835

[B161] Sander D. (2013). “Models of emotion: the affective neuroscience approach,” in *The Cambridge handbook of human affective neuroscience*, ed. Armony J. (Cambridge, MA: Cambridge University Press), 5–53. 10.1017/cbo9780511843716.003

[B162] Sander D. Grafman J. Zalla T. (2003). The human amygdala: an evolved system for relevance detection. *Rev. Neurosci.* 14 303–316. 14640318 10.1515/revneuro.2003.14.4.303

[B163] Sander D. Grandjean D. Scherer K. R. (2018). An appraisal-driven componential approach to the emotional brain. *Emot. Rev.* 10 219–231. 10.1177/1754073918765653

[B164] Särkkä S. Solin A. Nummenmaa A. Vehtari A. Auranen T. Vanni S. (2012). Dynamic retrospective filtering of physiological noise in BOLD fMRI: DRIFTER. *NeuroImage* 60 1517–1527. 10.1016/j.neuroimage.2012.01.067 22281675 PMC3303954

[B165] Satpute A. B. Lindquist K. A. (2019). The default mode network’s role in discrete emotion. *Trends Cogn. Sci.* 23 851–864. 10.1016/j.tics.2019.07.003 31427147 PMC7281778

[B166] Satpute A. B. Nook E. C. Narayanan S. Shu J. Weber J. Ochsner K. N. (2016). Emotions in “black and white” or shades of gray? How we think about emotion shapes our perception and neural representation of emotion. *Psychol. Sci.* 27 1428–1442. 10.1177/0956797616661555 27670663 PMC5111864

[B167] Sawahata Y. Komine K. Morita T. Hiruma N. (2013). Decoding humor experiences from brain activity of people viewing comedy movies. *PLoS One* 8:e81009. 10.1371/journal.pone.0081009 24324656 PMC3852249

[B168] Scherer K. R. (2004). Which emotions can be induced by music? What Are the Underlying Mechanisms? And How Can We Measure Them? *J. New Music Res.* 33 239–251. 10.1080/0929821042000317822

[B169] Scherer K. R. (2009). The dynamic architecture of emotion: evidence for the component process model. *Cogn. Emot.* 23 1307–1351. 10.1080/02699930902928969

[B170] Schilbach L. (2010). A second-person approach to other minds [Review of A second-person approach to other minds]. *Nat. Rev. Neurosci.* 11:449. 10.1038/nrn2805-c1 20485366

[B171] Schlochtermeier L. H. Pehrs C. Bakels J.-H. Jacobs A. M. Kappelhoff H. Kuchinke L. (2017). Context matters: anterior and posterior cortical midline responses to sad movie scenes. *Brain Res.* 1661 24–36. 10.1016/j.brainres.2016.12.013 27993532

[B172] Schnell K. Bluschke S. Konradt B. Walter H. (2011). Functional relations of empathy and mentalizing: an fMRI study on the neural basis of cognitive empathy. *NeuroImage* 54 1743–1754. 10.1016/j.neuroimage.2010.08.024 20728556

[B173] Schramm H. Wirth W. (2010). Exploring the paradox of sad-film enjoyment: the role of multiple appraisals and meta-appraisals. *Poetics* 38 319–335. 10.1016/j.poetic.2010.03.002

[B174] Schubert E. (2004). Modeling perceived emotion with continuous musical features. *Music Percept.* 21 561–585. 10.1525/mp.2004.21.4.561 33021500

[B175] Seth A. K. (2016). Infer yourself: interoception and internal “action” in conscious selfhood. *Behav. Brain Sci.* 39:e196. 10.1017/S0140525X15002265 28355805

[B176] Siegel E. H. Sands M. K. Van den Noortgate W. Condon P. Chang Y. Dy J. (2018). Emotion fingerprints or emotion populations? A meta-analytic investigation of autonomic features of emotion categories. *Psychol. Bull.* 144 343–393. 10.1037/bul0000128 29389177 PMC5876074

[B177] Simony E. Chang C. (2020). Analysis of stimulus-induced brain dynamics during naturalistic paradigms. *NeuroImage* 216:116461. 10.1016/j.neuroimage.2019.116461 31843711 PMC7418522

[B178] Simony E. Honey C. J. Chen J. Lositsky O. Yeshurun Y. Wiesel A. (2016). Dynamic reconfiguration of the default mode network during narrative comprehension. *Nat. Commun.* 7:12141. 10.1038/ncomms12141 PMC4960303 27424918

[B179] Skerry A. E. Saxe R. (2014). A common neural code for perceived and inferred emotion. *J. Neurosci.* 34 15997–16008. 10.1523/jneurosci.1676-14.2014 25429141 PMC4244468

[B180] Skerry A. E. Saxe R. (2015). Neural representations of emotion are organized around abstract event features. *Curr. Biol.* 25 1945–1954. 10.1016/j.cub.2015.06.009 26212878 PMC4824044

[B181] Smirnov D. Lachat F. Peltola T. Lahnakoski J. M. Koistinen O.-P. Glerean E. (2017). Brain-to-brain hyperclassification reveals action-specific motor mapping of observed actions in humans. *PLoS One* 12:e0189508. 10.1371/journal.pone.0189508 29228054 PMC5724834

[B182] Smirnov D. Saarimäki H. Glerean E. Hari R. Sams M. Nummenmaa L. (2019). Emotions amplify speaker-listener neural alignment. *Hum. Brain Mapp.* 40 4777–4788. 10.1002/hbm.24736 31400052 PMC6865790

[B183] Socher R. Perelygin A. Wu J. Chuang J. Manning C. D. Ng A. (2013). “Recursive deep models for semantic compositionality over a sentiment treebank,” in *Proceedings of the 2013 Conference on Empirical Methods in Natural Language Processing*, Seattle, 1631–1642.

[B184] Sonkusare S. Breakspear M. Guo C. (2019). Naturalistic stimuli in neuroscience: critically acclaimed. *Trends Cogn. Sci.* 23 699–714. 10.1016/j.tics.2019.05.004 31257145

[B185] Sonnemans J. Frijda N. H. (1994). The structure of subjective emotional intensity. *Cogn. Emot.* 8 329–350. 10.1080/02699939408408945

[B186] Speer N. K. Reynolds J. R. Swallow K. M. Zacks J. M. (2009). Reading stories activates neural representations of visual and motor experiences. *Psychol. Sci.* 20 989–999. 10.1111/j.1467-9280.2009.02397.x 19572969 PMC2819196

[B187] Stevenson R. A. Mikels J. A. James T. W. (2007). Characterization of the Affective Norms for English Words by discrete emotional categories. *Behav. Res. Methods* 39 1020–1024. 10.3758/bf03192999 18183921

[B188] Sznycer D. Cosmides L. Tooby J. (2017). Adaptationism carves emotions at their functional joints. *Psychol. Inq.* 28 56–62. 10.1080/1047840x.2017.1256132

[B189] Tarvainen J. Sjöberg M. Westman S. Laaksonen J. Oittinen P. (2014). Content-based prediction of movie style, aesthetics, and affect: data set and baseline experiments. *IEEE Trans. Multimedia* 16 2085–2098. 10.1109/tmm.2014.2357688

[B190] Tarvainen J. Westman S. Oittinen P. (2015). The way films feel: aesthetic features and mood in film. *Psychol. Aesthetics Creat. Arts* 9 254. 10.1037/a0039432

[B191] Taylor S. F. Phan K. L. Decker L. R. Liberzon I. (2003). Subjective rating of emotionally salient stimuli modulates neural activity. *NeuroImage* 18 650–659. 10.1016/s1053-8119(02)00051-4 12667842

[B192] Trost W. Frühholz S. Cochrane T. Cojan Y. Vuilleumier P. (2015). Temporal dynamics of musical emotions examined through intersubject synchrony of brain activity. *Soc. Cogn. Affect. Neurosci.* 10 1705–1721. 10.1093/scan/nsv060 25994970 PMC4666110

[B193] Tu P. C. Su T. P. Lin W. C. Chang W. C. Bai Y. M. Li C. T. (2019). Reduced synchronized brain activity in schizophrenia during viewing of comedy movies. *Sci. Rep.* 9 1–11. 31484998 10.1038/s41598-019-48957-w PMC6726596

[B194] Vaccaro A. G. Kaplan J. T. Damasio A. (2020). Bittersweet: the neuroscience of ambivalent affect. *Perspect. Psychol. Sci.* 15 1187–1199. 10.1177/1745691620927708 32758063

[B195] van der Meer J. N. Breakspear M. Chang L. J. Sonkusare S. Cocchi L. (2020). Movie viewing elicits rich and reliable brain state dynamics. *Nat. Commun.* 11:5004. 10.1038/s41467-020-18717-w 33020473 PMC7536385

[B196] Verduyn P. Van Mechelen I. Tuerlinckx F. Meers K. Van Coillie H. (2009). Intensity profiles of emotional experience over time. *Cogn. Emot.* 23 1427–1443. 10.1080/02699930902949031

[B197] Volynets S. Smirnov D. Saarimäki H. Nummenmaa L. (2020). Statistical pattern recognition reveals shared neural signatures for displaying and recognizing specific facial expressions. *Soc. Cogn. Affect. Neurosci.* 15 803–813. 10.1093/scan/nsaa110 33007782 PMC7543934

[B198] Wager T. D. Kang J. Johnson T. D. Nichols T. E. Satpute A. B. Barrett L. F. (2015). A Bayesian model of category-specific emotional brain responses. *PLoS Comput. Biol.* 11:e1004066. 10.1371/journal.pcbi.1004066 25853490 PMC4390279

[B199] Wallentin M. Nielsen A. H. Vuust P. Dohn A. Roepstorff A. Lund T. E. (2011). Amygdala and heart rate variability responses from listening to emotionally intense parts of a story. *NeuroImage* 58 963–973. 10.1016/j.neuroimage.2011.06.077 21749924

[B200] Walther D. Koch C. (2006). Modeling attention to salient proto-objects. *Neural Netw.* 19 1395–1407. 10.1016/j.neunet.2006.10.001 17098563

[B201] Waugh C. E. Shing E. Z. Avery B. M. (2015). Temporal dynamics of emotional processing in the brain. *Emot. Rev.* 7 323–329. 10.1177/1754073915590615

[B202] Westermann R. Spies K. Stahl G. Hesse F. W. (1996). Relative effectiveness and validity of mood induction procedures: a meta-analysis. *Eur. J. Soc. Psychol.* 26 557–580. 10.1002/(sici)1099-0992(199607)26:4<557::aid-ejsp769>3.0.co;2-4

[B203] Witkower Z. Tracy J. L. (2019). Bodily communication of emotion: evidence for extrafacial behavioral expressions and available coding systems. *Emot. Rev. J. Int. Soc. Res. Emot.* 11 184–193. 10.1177/1754073917749880

[B204] Yang W. Makita K. Nakao T. Kanayama N. Machizawa M. G. Sasaoka T. (2018). Affective auditory stimulus database: an expanded version of the International Affective Digitized Sounds (IADS-E). *Behav. Res. Methods* 50 1415–1429. 10.3758/s13428-018-1027-6 29520632

[B205] Yao Z. Wu J. Zhang Y. Wang Z. (2017). Norms of valence, arousal, concreteness, familiarity, imageability, and context availability for 1,100 Chinese words. *Behav. Res. Methods* 49 1374–1385. 10.3758/s13428-016-0793-2 27553483

[B206] Young C. B. Raz G. Everaerd D. Beckmann C. F. Tendolkar I. Hendler T. (2017). Dynamic shifts in large-scale brain network balance as a function of arousal. *J. Neurosci.* 37 281–290. 10.1523/jneurosci.1759-16.2017 28077708 PMC6596574

[B207] Zaki J. Weber J. Bolger N. Ochsner K. (2009). The neural bases of empathic accuracy. *Proc. Natl. Acad. Sci. U.S.A.* 106 11382–11387. 10.1073/pnas.0902666106 19549849 PMC2708723

