# Supplementary material for: Naturalistic Stimuli in Affective Neuroimaging: A Review
Source: Front Hum Neurosci. 2021 Jun 17;15:675068. doi: 10.3389/fnhum.2021.675068 (PMC8245682; doi:10.3389/fnhum.2021.675068)
Supplement: Supplementary file 1 [file Table_1.docx]

**SUPPLEMENTARY MATERIAL**

**Table S1.** Summary of brain regions correlating with emotion features. The regions are organized by anatomical regions. (+) and (-) refer to unambiguous positive and negative correlations, respectively. For study indexes, refer to Table 1.

|  | **Stimulus features** | | | **Observer features** | | | | | |  |
| --- | --- | --- | --- | --- | --- | --- | --- | --- | --- | --- |
| **Region** | **Low-level** | **Object-**  **level** | **Portrayed emotions** | **Elicitation** | **Intero-**  **ception** | **Behavior** | **Affective dimensions** | **Emotion categories** | **Emotional alignment** | **Studies** |
| ***Subcortical nuclei*** | | | | | | | | | | |
| Amygdala | Auditory: RMS, dissonance | Lexical arousal |  |  | Startle |  | Valence, arousal (+) | Amusement, anger, fear, sadness | Similarity of valence and arousal | [1, 2, 3, 9, 20, 25, 28, 30, 32] |
| Hippocampus |  | Lexical arousal |  |  |  |  | Valence (+) | Amusement | Similarity of arousal | [2, 10, 28, 32] |
| Thalamus | Auditory: centroid, brightness, zero-crossing, tempo |  |  |  | Startle |  | Valence, arousal | Amusement, sadness. Fear (-) |  | [2, 3, 20, 25, 28, 30] |
| Caudate nucleus |  | Lexical arousal |  |  | Startle |  | Valence (-), arousal (+) | Amusement. Fear (-) |  | [2, 20, 30, 32] |
| Nucleus accumbens | Auditory: centroid, brightness, zero-crossing, RMS, dissonance (-) |  |  |  |  |  |  | Amusement |  | [10, 30] |
| Putamen | Auditory: centroid, brightness, zero-crossing, RMS, tempo, pulse clarity |  |  |  | Startle |  | Valence (-) |  |  | [3, 20, 29, 30] |
| Substantia nigra |  |  |  |  | Startle |  |  |  |  | [20] |
| ***Cingulate gyrus*** | | | | | | | | | | |
| Anterior cingulate | Auditory: centroid, brightness, zero-crossing, entropy, tempo. Auditory: sound energy (-) | Lexical arousal (-) |  |  | Startle |  | Valence, arousal |  | Similarity of valence | [18, 20, 26, 28, 29, 30, 32] |
| Middle cingulate | Auditory: sound energy (-) |  |  |  | Startle |  |  |  |  | [20, 27] |
| Posterior cingulate | Auditory: sound energy (-) | Lexical arousal |  |  | Startle |  | Valence, arousal (-) | Amusement, sadness |  | [2, 3, 20, 27, 30] |
| ***Insular gyrus*** | | | | | | | | | | |
| Insula | Auditory: RMS, dissonance, tempo.  Auditory: sound energy (-) |  |  |  | Heart rate, startle |  | Valence (+), arousal (-) | Amusement, anger. Fear (-) |  | [1, 10, 13, 18, 20, 27, 29, 30, 32] |
| ***Frontal lobe*** | | | | | | | | | | |
| Superior frontal gyrus (dorsomedial) | Auditory: dissonance |  |  |  |  |  | Valence, arousal | Amusement, fear, sadness, suspense |  | [2, 3, 10, 11, 17, 20, 26, 28, 29, 30, 32, 33] |
| Superior frontal gyrus (dorsolateral) | Auditory: centroid, brightness, zero-crossing, tempo |  |  |  |  |  | Arousal (+) |  |  | [26, 29, 30] |
| Middle frontal gyrus (anterior) | Auditory: tempo | Lexical arousal (-) |  |  |  |  | Valence (+) |  |  | [8, 10, 29, 32] |
| Middle frontal gyrus (dorsolateral) | Auditory: centroid, brightness, zero-crossing. Auditory: sound energy (-) | Lexical valence (+) Lexical arousal (-) |  | Expected humor |  |  | Valence, arousal | Amusement |  | [2, 16, 18, 26, 27, 28, 30, 32] |
| Inferior frontal gyrus | Auditory: centroid, brightness, zero-crossing,entropy, event density, RMS, dissonance, tempo, sound energy |  | Audience laughter | Expected humor | Heart rate (HF-HRV), startle |  | Valence. Arousal (+) | Amusement, fear, sadness, suspense |  | [1, 2, 3, 5, 10, 11, 20, 25, 27, 28, 30, 32, 33] |
| Orbital gyrus (ventromedial) | Auditory: tempo | Lexical arousal (-) |  |  |  | Facial behavior | Valence (+), arousal | Amusement, anger, fear, multiple emotions |  | [3, 10, 13, 20, 24, 25, 26, 30, 32] |
| Precentral gyrus | Auditory: entropy, event density, tempo | Lexical valence (+) Lexical arousal (-) | Audience laughter |  | Startle | Facial motion | Arousal (+) | Amusement, fear, suspense | Similarity of valence | [3, 16, 20, 25, 28, 29, 30, 32, 33] |
| ***Temporal lobe*** |  |  |  |  |  |  |  |  |  |  |
| Temporal lobe |  |  |  | Expected humor |  | Facial motion |  |  |  | [5] |
| Superior temporal gyrus | Auditory: centroid, brightness, zero-crossing, entropy, event density, RMS, dissonance, tempo, sound energy | Lexical valence (+) Lexical arousal (-) |  |  | Startle |  | Valence, arousal | Amusement, sadness | Similarity of valence | [2, 3, 8, 10, 20, 25, 26, 27, 28, 29, 30, 32] |
| Middle temporal gyrus |  | Lexical valence (-) |  | Expected humor | Startle |  | Valence, arousal | Amusement, sadness | Similarity of valence | [1, 2, 3, 8, 16, 20, 25, 26, 32] |
| Inferior temporal gyrus |  |  |  |  |  |  | Valence (-). Arousal (+) | Amusement |  | [3, 8] |
| Fusiform gyrus | Auditory: sound energy |  |  |  | Startle |  | Valence (+), arousal (+) | Amusement, fear, sadness | Similarity of arousal | [2, 3, 8, 10, 20, 27, 28] |
| Parahippocampal gyrus | Auditory: sound energy (-) | Non-emotional auditory track, lexical valence (+)  Lexical arousal (-) |  |  | Startle |  | Arousal (+) | Amusement, sadness. Fear (-) |  | [2, 3, 8, 10, 20, 27, 28] |
| Middle temporal sulcus |  | Lexical arousal |  |  |  |  |  |  |  | [32] |
| Posterior superior temporal sulcus |  |  |  |  |  |  | Valence, arousal (+) | Suspense |  | [28, 33] |
| Temporo-parietal junction |  | Lexical valence |  |  |  |  | Valence (+), arousal (-) | Suspense, multiple emotions |  | [15, 25, 32, 33] |
| ***Parietal lobe*** |  |  |  |  |  |  |  |  |  |  |
| Parietal lobe |  |  | Audience laughter |  |  | Facial motion |  | Amusement |  | [5] |
| Superior parietal lobule |  |  |  |  |  |  | Valence, arousal (+) | Amusement | Similarity of valence | [3, 10, 16, 28] |
| Inferior parietal lobule | Auditory: tempo | Lexical valence (-)  Lexical arousal. |  |  |  |  | Valence (+), arousal | Amusement | Similarity of valence | [3, 8, 10, 16, 25, 26, 28, 29, 32] |
| Precuneus | Auditory: entropy | Lexical valence (+)  Lexical arousal (-) |  |  | Startle |  | Valence, arousal | Fear, sadness | Similarity of valence | [2, 3, 11, 17, 20, 25, 26, 28, 32] |
| Postcentral gyrus | Auditory: tempo |  |  |  | Heart rate (HF-HRV) |  | Valence, arousal (+) | Amusement | Similarity of arousal | [3, 8, 16, 27, 28, 29, 30] |
| ***Occipital lobe*** |  |  |  |  |  |  |  |  |  |  |
| Occipital lobe |  |  |  |  |  | Facial motion |  |  |  | [5] |
| Striate cortex (V1) |  | Lexical arousal |  |  |  |  | Arousal (+) |  |  | [3, 32] |
| Prestriate cortex |  |  |  |  |  |  |  | Amusement | Similarity of arousal | [10, 28] |
| Extrastriate cortex |  | Lexical valence (+) |  |  |  |  |  |  |  | [32] |
| Cuneus gyrus |  |  |  |  | Startle |  | Arousal (+) | Fear, sadness |  | [2, 3, 11, 20, 29] |
| Lingual gyrus | Auditory: tempo |  |  |  | Startle |  | Valence, Arousal (-) | Amusement, fear |  | [8, 10, 20, 26, 28, 29] |
| Inferior occipital gyrus |  |  |  |  |  |  |  | Sadness |  | [2] |
| Middle occipital gyrus | Auditory: sound energy | Non-emotional auditory track |  |  | Startle |  | Valence (-), Arousal (+) | Fear, sadness |  | [2, 3, 20, 27, 29] |
| Superior occipital gyrus |  |  |  |  |  |  | Valence |  |  | [3, 26] |
| Lateral occipital cortex |  | Valence of pictures |  |  |  |  | Valence (-) | Amusement |  | [8, 17, 28] |
| ***Cerebellum*** |  |  |  |  |  |  |  |  |  |  |
| Cerebellum |  | Lexical valence, lexical arousal |  |  | Startle |  | Valence (-), arousal | Amusement, fear |  | [3, 8, 20, 26, 32] |
