# Supplementary material for: Naturalistic Stimuli in Affective Neuroimaging: A Review
Source: Front Hum Neurosci. 2021 Jun 17;15:675068. doi: 10.3389/fnhum.2021.675068 (PMC8245682; doi:10.3389/fnhum.2021.675068)
Supplement: Supplementary file 2 [file Table_2.docx]

**SUPPLEMENTARY MATERIAL**

**Table S2.** Summary of brain networks correlating with emotion features. (+) and (-) refer to unambiguous positive and negative correlations, respectively. For study indexes, refer to Table 1.

|  | **Stimulus features** | | | **Observer features** | | | | | |  |
| --- | --- | --- | --- | --- | --- | --- | --- | --- | --- | --- |
| **Functional network** | **Low-level** | **Object-**  **level** | **Portrayed emotions** | **Elicitation** | **Intero-**  **ception** | **Behavior** | **Affective dimensions** | **Emotion categories** | **Emotional alignment** | **Studies** |
| Auditory |  | Spoken dialogue, positive and negative scenes | Positive and negative facial expressions |  | Pupil dilation,  heart rate (-) |  |  | Enjoyment |  | [22, 31] |
| Default mode | Scene cuts | Aversive scenes, positive and negative scenes, spoken dialogue | Negative facial expressions |  | Heart rate, pupil dilation |  | Valence | Enjoyment, surprise |  | [3, 12, 14, 22, 23, 31] |
| Dorsal attention |  |  |  | Anticipation of aversive scenes |  |  | Arousal |  |  | [3, 14] |
| Executive |  | Positive scenes | Positive facial expressions |  | Pupil dilation |  |  |  |  | [22] |
| Frontal |  |  |  |  |  |  |  | Enjoyment, sadness |  | [4, 31] |
| Fronto-parietal |  |  |  | Anticipation of aversive scenes |  |  | Arousal | Suspense |  | [6] |
| Language |  | Spoken dialogue, positive and negative scenes | Positive and negative facial expressions |  | Pupil dilation,  heart rate (-) |  |  |  |  | [22] |
| Limbic | Auditory: RMS |  |  |  | Heart rate (HF-HR) |  |  | Sadness |  | [4, 7, 31] |
| Salience |  | Emotional auditory track, positive and negative scenes | Negative facial expressions |  | Heart rate, pupil dilation |  |  |  |  | [12, 22, 27] |
| Somatomotor |  | Aversive scenes |  |  |  |  |  |  |  | [14] |
| Striatal | Auditory: brightness, RMS |  |  |  |  |  |  | Enjoyment, sadness |  | [31] |
| Theory of mind |  |  |  |  |  |  |  | Sadness |  | [7] |
| Visual | Scene cuts | Positive scenes | Positive facial expressions |  | Pupil dilation |  | Arousal |  |  | [3, 22] |
